# Synthetic reactions driven by electron-donor–acceptor (EDA) complexes

**DOI:** 10.3762/bjoc.17.67

**Published:** 2021-04-06

**Authors:** Zhonglie Yang, Yutong Liu, Kun Cao, Xiaobin Zhang, Hezhong Jiang, Jiahong Li

**Affiliations:** 1 School of Life Science and Engineering, Southwest Jiaotong University, Chengdu 610031, China; 2 Irradiation Preservation Key Laboratory of Sichuan Province, Radiation Chemistry Department, Sichuan Institute of Atomic Energy, Chengdu 610100, China

**Keywords:** EDA complex, electron acceptor, electron donor, radical, visible light

## Abstract

The reversible, weak ground-state aggregate formed by dipole–dipole interactions between an electron donor and an electron acceptor is referred to as an electron-donor–acceptor (EDA) complex. Generally, upon light irradiation, the EDA complex turns into the excited state, causing an electron transfer to give radicals and to initiate subsequent reactions. Besides light as an external energy source, reactions involving the participation of EDA complexes are mild, obviating transition metal catalysts or photosensitizers in the majority of cases and are in line with the theme of green chemistry. This review discusses the synthetic reactions concerned with EDA complexes as well as the mechanisms that have been shown over the past five years.

## Review

### Introduction

**Scheme 1:**
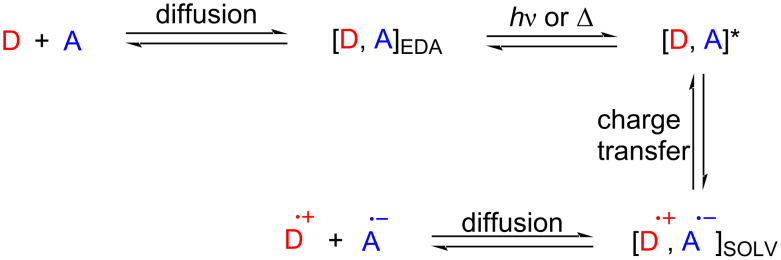
The electron transfer process in EDA complexes.

Electron transfer (ET) is a very common occurrence in the field of natural science, including photochemical, electrochemical, and enzymatic reactions and even major organic synthesis. From 1950 to 1952, Mulliken suggested an electron transfer hypothesis that could more precisely explain electron transfer phenomena based on the electron-donor–acceptor (EDA) complex [1–3]. Significantly, a broad absorption peak unrelated to the structure, called charge-transfer band, is typically located in the visible region of the UV–vis spectrum [4], which manifests the color variability of the mixed solution of the electron donor (D) and electron acceptor (A). The two components A and D may not absorb visible light, but the resulting EDA complex does [5]. If the EDA complex is irradiated with a particular wavelength (or heated to a corresponding temperature), the complex could be excited to the state [D, A]*, causing electron transfer and forming a pair of radical ions trapped in the solvent cage. The pair of radical ions escapes the solvent cage by diffusion to give radical ions, which could initiate chemical reactions or reverse electron transfer (Scheme 1) [6]. The continuously increasing demand for sustainable synthesis has encouraged chemists to pursue more efficient methods to manufacture fine and usable chemicals [7]. The reactions that EDA complexes participate in have been shown to be an enormous success, mainly due to the fact that they obviate photoredox catalysts or transition metal catalysts in the vast majority cases. Moreover, in line with the theme of green chemistry, light is the sole external energy source in EDA complex pathways. Except for the pioneering research on EDA complexes in the 20th century, there was not much progress in the follow-up. Until the past few years, EDA-complex photochemistry has attracted more and more chemists and provided new opportunities for synthetic chemistry [8]. Moreover, diverse photocatalyst-free photochemical reactions have been employed to construct carbon–carbon and carbon–heteroatom bonds [9]. Among these methods, the product formations by aid of EDA complexes are the simplest and most rapid way. In this review, the focus lies on cyclization reactions, C–C-, C–S-, C–B-, C–N-, C–P-, C–O-, and C–H bond formation, primarily summarizing the chemical reaction step involving the EDA complex (Table 1) as well as the underlying mechanisms that have appeared over the last five years.

**Table 1:** The EDA complexes discussed in this review.

EDA complex	Ref.	EDA complex	Ref.	EDA complex	Ref

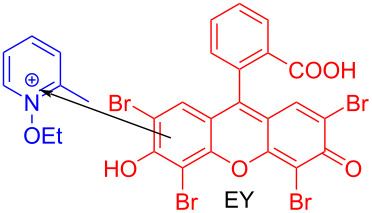	[10]	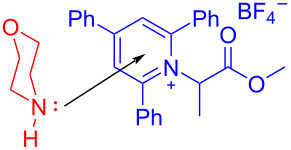	[11]	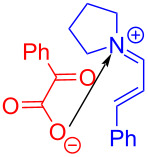	[12]
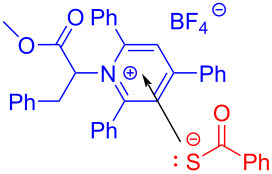	[13]	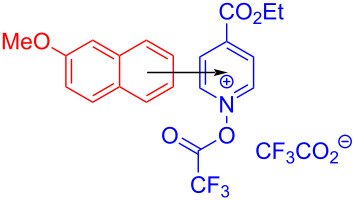	[14]	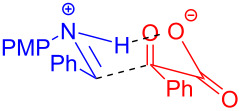	[15]
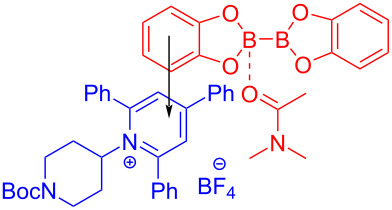	[16]	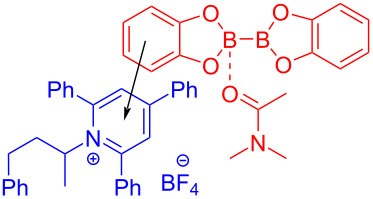	[17]	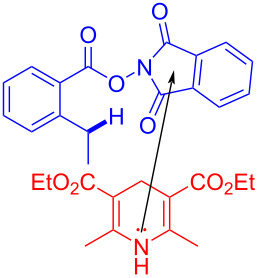	[18]
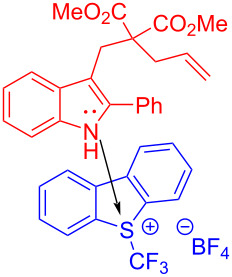	[19]	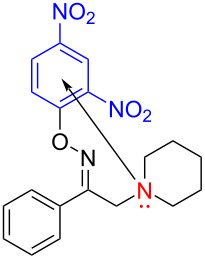	[20]	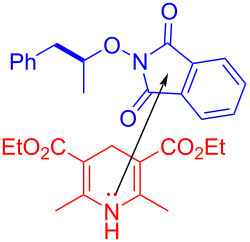	[21]
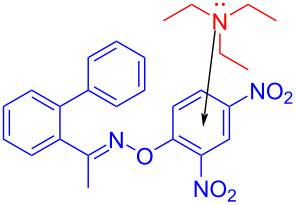	[22]	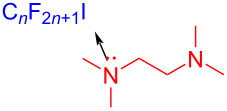	[23]	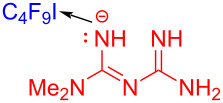	[24]
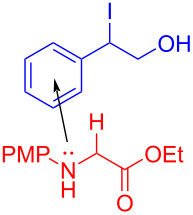	[25]	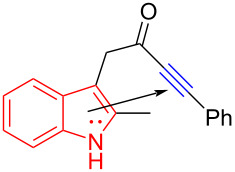	[26]	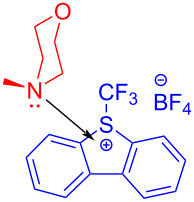	[27]
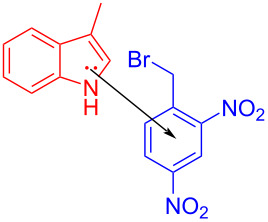	[28]	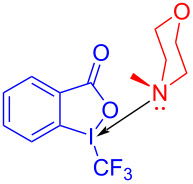	[29–30]	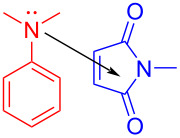	[31]
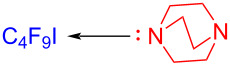	[32]	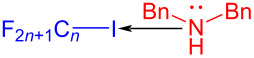 *n* = 1,3,4,6–8	[33]	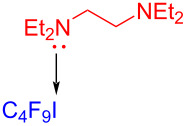	[34]
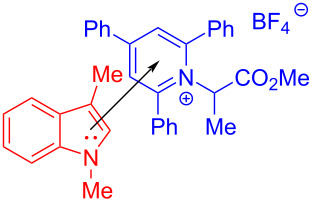	[11]	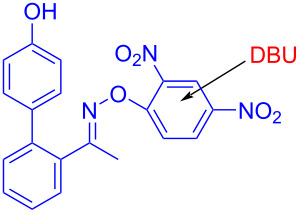	[35]	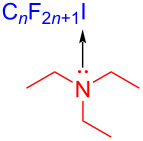 *n* = 1–4,6	[36]
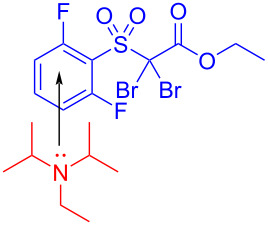	[37]	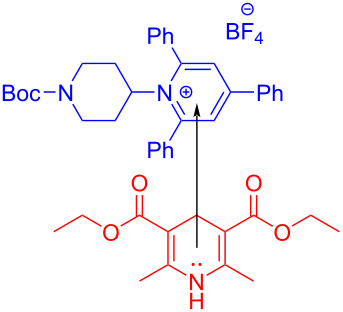	[38]	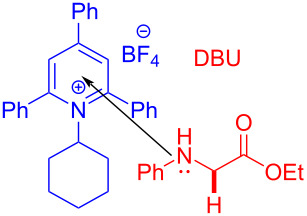	[39]
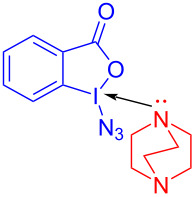	[40]	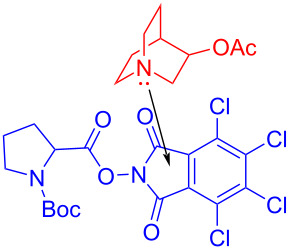	[41]	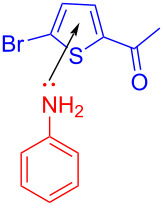	[42]
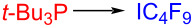	[43]	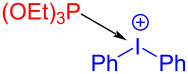	[44]	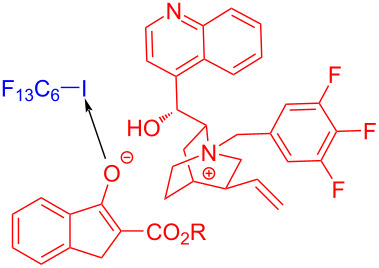	[45]
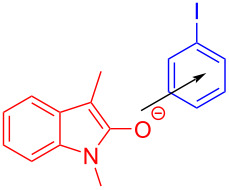	[46]	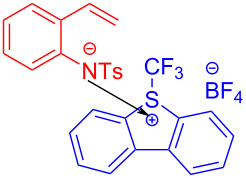	[47]	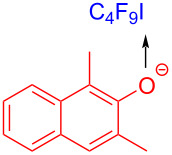	[48]
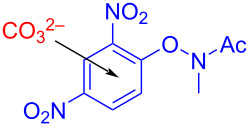	[49]	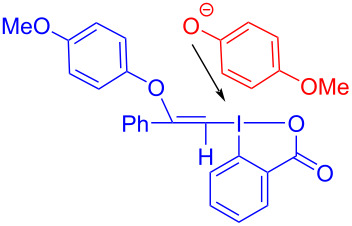	[50]	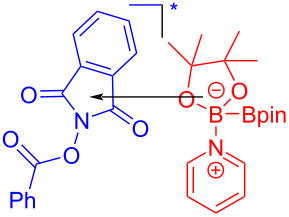	[51]
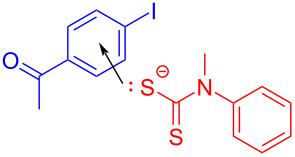	[52]	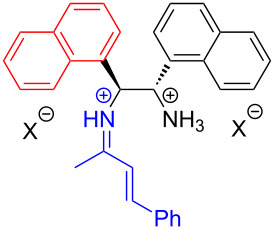	[53]	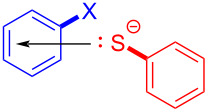	[54]
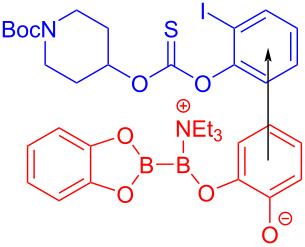	[55]	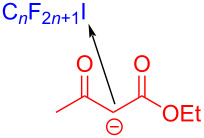 *n* = 2,3,4,6,8,10	[56]	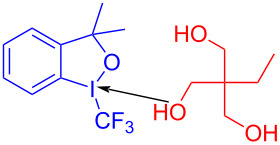	[57]
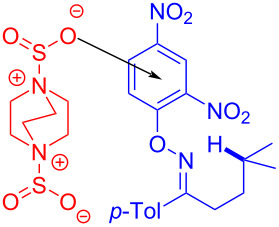	[58]				

### Cyclization reactions

In the pharmaceutical industry, retail drugs with a heterocyclic composition have exceeded 60% of the market volume. Hence, cyclization reaction innovation seems to be a requisite for pharmaceutical industry and human health. As an outstanding way, free-radical cascade reactions could efficiently construct various carbocycles and heterocycles with multifarious structures and complexity [59–61]. Centered on this context, we give a clear overview on a variety of novel cyclization reactions initiated by EDA complexes from the recent years.

**Scheme 2:**
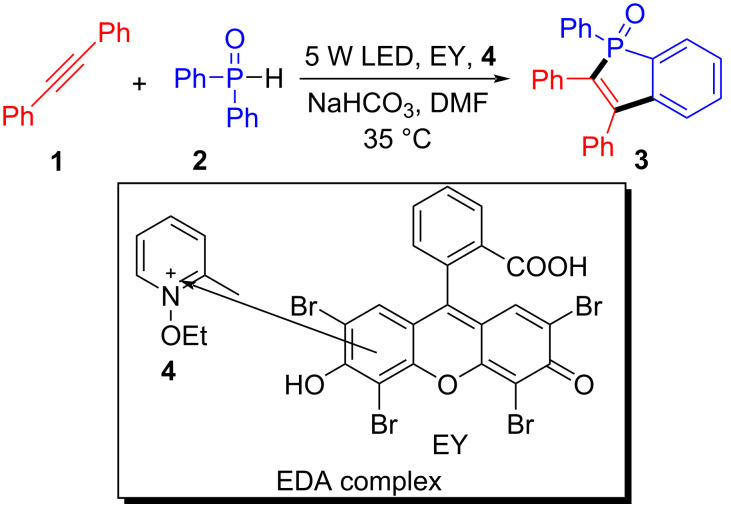
Synthesis of benzo[*b*]phosphorus oxide **3** initiated by an EDA complex.

In 2016, Lakhdar and colleagues [10] obtained the target product **3** with LED (5 W) irradiation of a solution containing arylphosphine oxide **2**, alkynes **1**, eosin Y (EY, 4 mol %), *N*-ethoxy-2-methylpyridinium (**4**), and sodium bicarbonate in DMF (Scheme 2). As a distinct example of EDA complexes, the process efficiency depends on the association of eosin Y and oxidant **4** to a donor–acceptor EY–**4** ground-system complex (high reactivity). Due to the ability of aryl groups to stabilize the formed alkenyl radical, this protocol could control regioselectivity efficiently with unsymmetrical alkynes. In addition, EPR spectroscopy shows that phosphono radicals could proceed throughout the reaction.

**Scheme 3:**
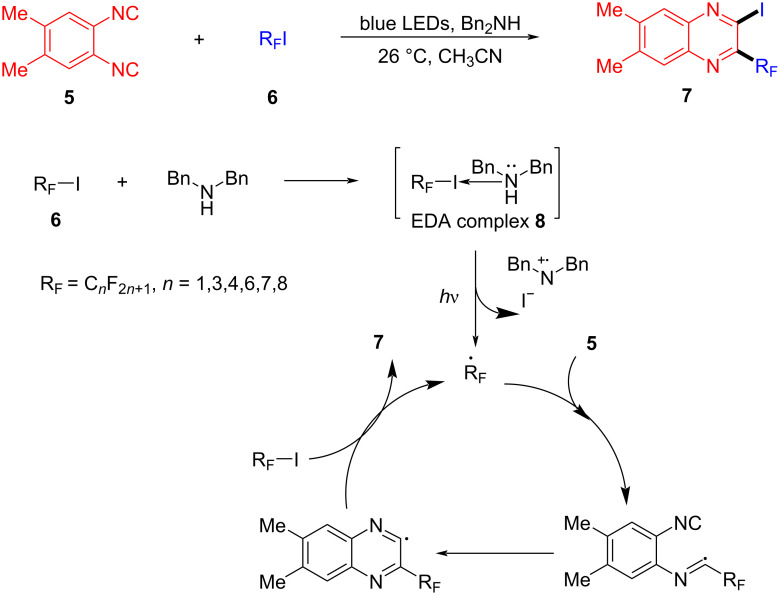
Mechanism of the synthesis of quinoxaline derivative **7**.

A halogen bond (XB) is a noncovalent interaction formed between a halogen atom and a neutral or negatively charged Lewis base. It is a kind of weak intermolecular interaction analogous to a hydrogen bond and basically can be considered as a specific EDA complex [62]. In 2016, Yu and colleagues [33] employed perfluoroalkyl iodide **6** as halogen-bond donor (electron acceptor) and the organic base dibenzylamine as the halogen-bond acceptor (electron donor) to form the XB complex **8**, and then a fluoroalkyl radical was given via visible-light-induced single-electron-transfer process. 1,2-Diisocyanato-4,5-xylene (**5**) was able to capture the fluoroalkyl radical, eventually providing the quinoxaline derivative **7** (Scheme 3).

**Scheme 4:**
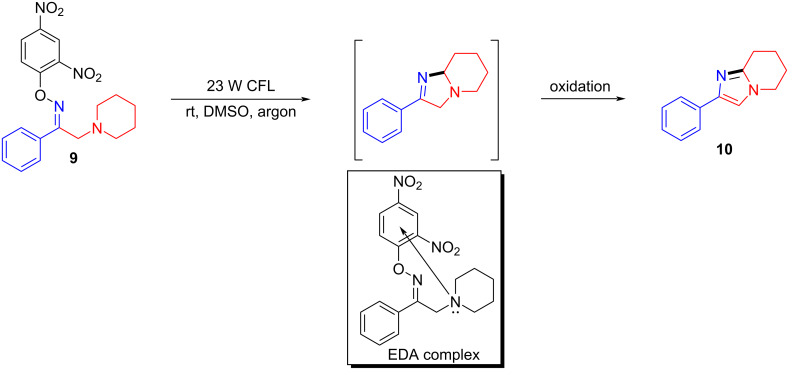
Synthesis of imidazole derivative **10** initiated by an EDA complex.

In 2017, Fu and colleagues [20] selected 1-phenyl-2-(piperidin-1-yl)ethanone *O*-(2,4-dinitrophenyl)oxime (**9**) as substrates under 23 W CFL (compact fluorescent lamp) irradiation, affording the desired imidazole derivative **10** by utilizing DMSO as the solvent at room temperature (Scheme 4). It is worth noting that, unlike most reported intermolecular electron-transfer via an EDA complex pathway, this approach transfers electrons from the electron-rich tertiary amine nitrogen atom to the electron-deficient benzene ring, achieving intramolecular electron transfer. Selective C–H-functionalization also includes no catalysts, oxidants, additives, acids and bases, which is of great significance in the synthesis and application of N-heterocyclic compounds.

**Scheme 5:**
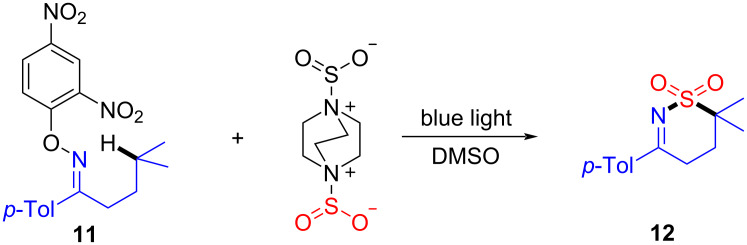
Synthesis of sulfamoylation product **12** initiated by an EDA complex.

In 2017, Wu and colleagues [58] uncovered the use of the bis(sulfur dioxide) adduct of DABCO, 1,4-diazabicyclo[2.2.2]octane·(SO_2_)_2_, as sulfone source in the EDA complex formation by 4-methyl-1-(*p*-tolyl)pentan-1-one *O*-(2,4-dinitrophenyl)oxime (**11**) towards the aminosulfonylation, employing blue light as irradiation source (Scheme 5). It has to be said that nitrogen radicals played a coordinating role in the sulfonation step. Additionally, to verify the applicability of this approach, 1*H*-benzo[*d*][1,2]thiazine 2,2-dioxides have been prepared successfully.

**Scheme 6:**
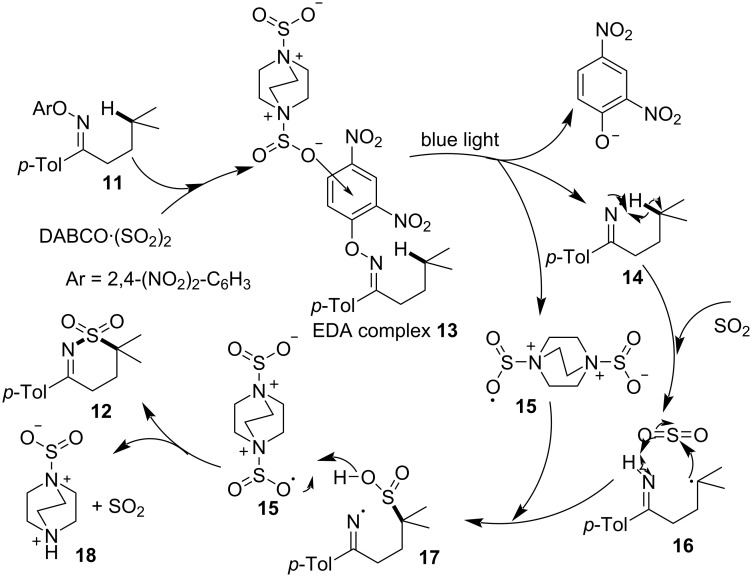
Mechanism of the synthesis of sulfamoylation product **12**.

A possible reaction mechanism for this transformation is as follows (Scheme 6): Firstly, *O*-aryloxime **11** forms EDA complex **13** by action of DABCO·(SO_2_)_2_ and then undergoes light-promoted single-electron transfer, affording the 2,4-dinitrophenol anion, nitrogen radical **14**, and radical **15**, respectively. 1,5-HAT (hydrogen atom transfer) occurs in nitrogen radical **14** to give radical **16**, which further transforms to radical **17** after the addition of sulfur dioxide. Finally, HAT happens between **15** and **17**, yielding quaternary ammonium salt **18** and product **12**, respectively.

**Scheme 7:**
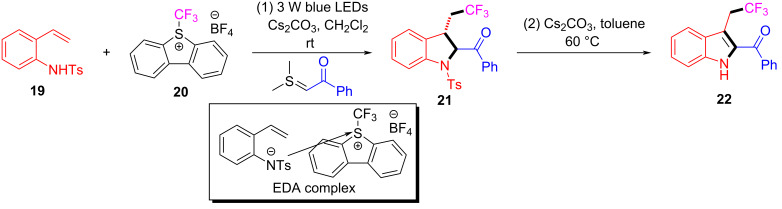
Synthesis of indole derivative **22** initiated by an EDA complex.

In 2017, Chen and colleagues [47] accomplished the cyclization through the EDA complex formed by *N*-tosyl-2-vinylaniline (**19**) and the Umemoto reagent **20** (CF_3_ radical source) in CH_2_Cl_2_ under blue LED irradiation. In the presence of base, **21** was produced with 98% yield after degassing. Along with straightforward posttreatment, the corresponding reduction product **22** can be afforded easily (Scheme 7). This procedure offers a novel cyclization method with bifunctionalization, causing a multicomponent reaction of vinylaniline, halide, and sulfonylate to give corresponding indole derivatives. Furthermore, a wide variety of applicable substrates and good functional-group tolerance are provided by this approach, yielding multiple indole analogues with biological activity.

**Scheme 8:**
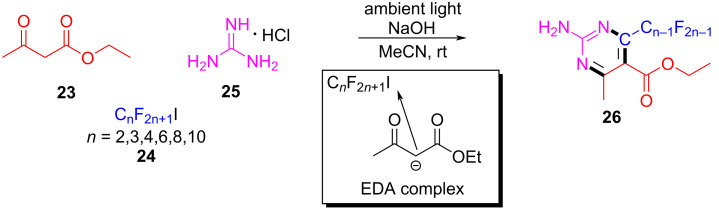
Synthesis of perfluoroalkylated pyrimidines **26** initiated by an EDA complex.

In 2017, Liang and Bi [56] reported a visible-light-induced three-component cyclization of ethyl acetoacetate (**23**), perfluoroalkyl iodides **24**, and guanidine hydrochloride (**25**) via a halogen-bond adduct. The first light-promoted three-component reaction has been realized by a halogen-bond adduct, forming perfluoroalkylated pyrimidines **26** (Scheme 8). A variety of perfluorinated chains were assembled with methylene compounds and guanidines or amidines, giving the corresponding perfluoroalkylated pyrimidines in good to excellent yield.

**Scheme 9:**
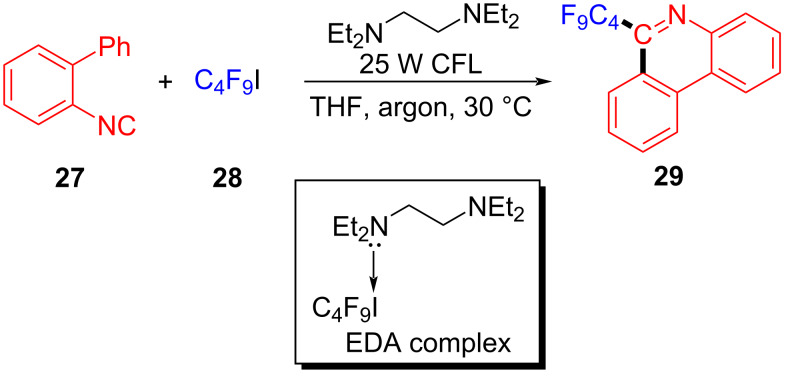
Synthesis of phenanthridine derivative **29** initiated by an EDA complex.

In 2017, Chen and colleagues [34] prepared the phenanthridine derivative **29** with CFL (25 W) irradiation of a solution containing **27**, perfluoroalkyl iodide **28**, amine additive *N,N,N´,N´*-tetraethylethylenediamine (TEEDA) in THF (Scheme 9). TEEDA and perfluoroalkyl iodide form a halogen-bond adduct, and then light-induced electron transfer happens in order to give a perfluoroalkyl radical. The protocol can realize alkene- and alkyne iodide perfluoroalkylation and C–H perfluoroalkylation of electron-rich heteroaromatic hydrocarbons, providing a novel protocol for the synthesis of perfluoroalkyl-substituted phenanthridines.

**Scheme 10:**
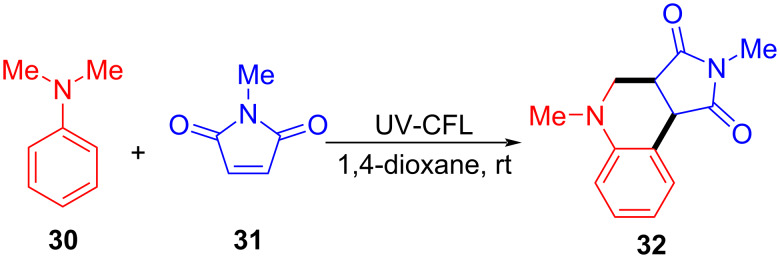
Synthesis of *cis*-tetrahydroquinoline derivative **32** initiated by an EDA complex.

**Scheme 11:**
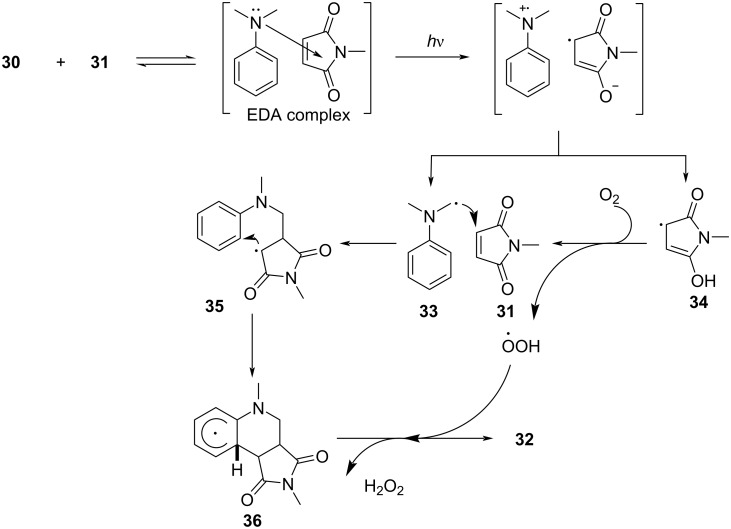
Mechanism of the synthesis of *cis*-tetrahydroquinoline derivative **32**.

In 2018, Sundén and Hsu [31] proposed a method of adding an α-aminoalkyl radical to maleimide via an EDA complex based on previous work (Scheme 10). The corresponding products can be given in good yield by modifying substituents on the *N*-alkyl moiety in **31** or *N*,*N*-dimethylaniline (**30**). This approach utilizes *N*,*N*-dimethylaniline (**30**) as electron donor and *N*-methylmaleimide (**31**) as electron acceptor to form an EDA complex, so that single-electron transfer occurs under ultraviolet-light irradiation. Subsequently, intermolecular proton transfer takes place, giving radicals **33** and **34**. Radical **34** is quenched by oxygen, and radical **33** attacks **31** in order to form radical **35**. Intermediate **36** is achieved by cyclization of radical **35**, followed by hydrogen-atom removal, providing the *cis*-tetrahydroquinoline **32** (Scheme 11). It is worth noting that the EDA complex not only undergoes charge transfer but also proton transfer in this approach. The optimization experiment showed that the wavelength of the light source must overlap with the absorption spectrum of the EDA complex. Most importantly, given that the best yield was achieved when the molar concentration of **30** was 7 times that of **31**, the concentration of the EDA complex was essential for a high reaction rate.

**Scheme 12:**
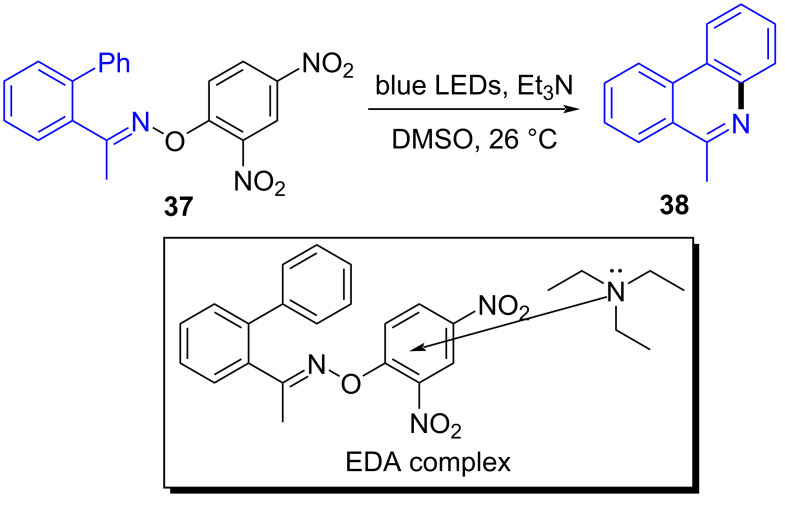
Synthesis of phenanthridine derivative **38** initiated by an EDA complex.

In 2018, Yu and colleagues [22] discovered a method that employed *O*-aryloxime **37** and triethylamine as substrates at room temperature and blue-light irradiation to give phenanthridine **38** (also including quinoline products, Scheme 12). In this way, a nitrogen-centered radical was given via the EDA complex that was initiated by single-electron transfer, accomplishing the synthesis of a variety of highly functionalized nitrogen-containing aromatics with excellent yield.

**Scheme 13:**
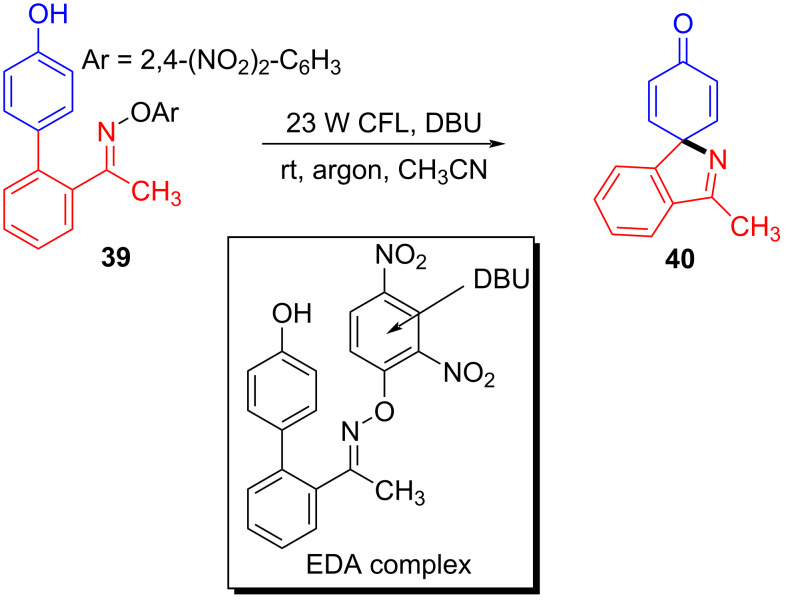
Synthesis of spiropyrroline derivative **40** initiated by an EDA complex.

In 2019, Fu and colleagues [35] received the target product **40** with 23 W CFL irradiation of a solution containing 1-(4′-hydroxy-[1,1′-biphenyl]-2-yl)ethanone *O*-(2,4-dinitrophenyl)oxime (**39**) and 1,8-diazabicyclo[5.4.0]undec-7-ene (DBU) in CH_3_CN (Scheme 13). Ready-made DBU serves two roles: base and electron donor. Furthermore, due to the commercially available material and wide range of substrates, this approach has major significance for drug scaffold methodologies, providing useful strategy for the synthesis of spiropyrrolines.

**Scheme 14:**
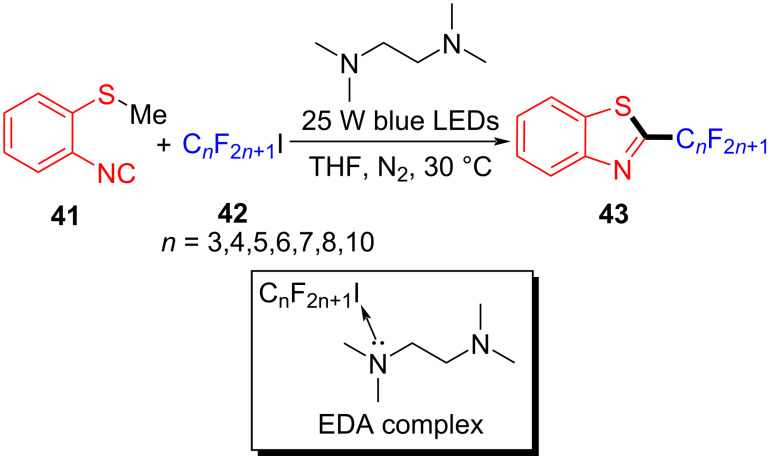
Synthesis of benzothiazole derivative **43** initiated by an EDA complex.

In 2019, Yu and colleagues [23] reported the utilization of tetramethylethylenediamine (TMEDA) as additive in the EDA complex formation with perfluoroalkyl iodides **42** to afford 2-perfluoroalkylbenzothiazole products **43**, employing blue LEDs (25 W) as irradiation source (Scheme 14). Notably, as (2-isocyanophenyl)(methyl)selane was exploited instead of substrate **41**, new fluoroalkylbenzoselenazole derivatives with biological potential could also be given successfully. Furthermore, several perfluoroalkyl iodides IC*_n_*F_2_*_n_*_+1_ (*n* = 3–8,10) and four other fluoroalkyl iodides, including ICF(CF_3_)_2_, ICF_2_COOEt, ICF_2_CF_2_Cl, and ICF_2_CF_2_Br were reacted smoothly with **41**, affording the corresponding products in good to excellent yield. The protocol conforms to the characteristics of green and environmental conservation, having a reaction time of only 1 hour, achieving the purpose of saving energy.

**Scheme 15:**
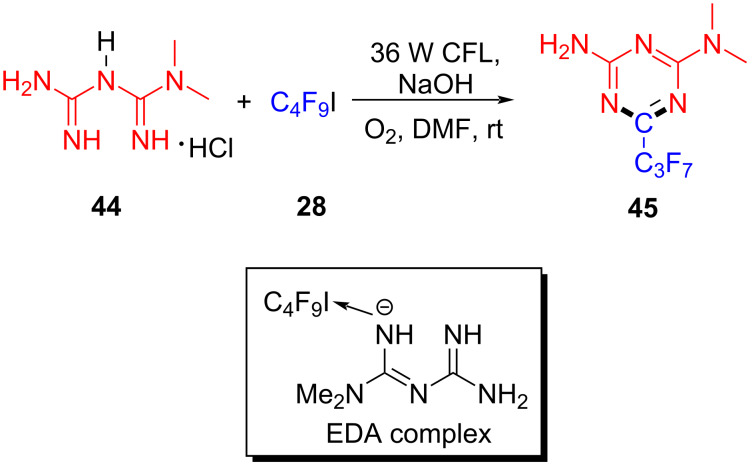
Synthesis of perfluoroalkyl-*s*-triazine derivative **45** initiated by an EDA complex.

In 2019, Liang and colleagues [24] reported a method for preparing perfluoroalkyl-*s*-triazine via visible-light-promoted [5 + 1] cyclization initiated by an EDA complex. Perfluoroalkyl-*s*-triazine derivative **45** was synthesized by the reaction of biguanidine derivative **44** and perfluoroalkyl iodide **28** in the presence of sodium hydroxide (Scheme 15). Considering that oxygen played an indispensable role in the process, the authors supposed that it may facilitate single-electron transfer between biguanidine anion and perfluoroalkyl halide. By constructing two C–N bonds at the same time, the triazine heterocyclic structure that is commonly utilized in medical and material fields was accomplished by [5 + 1] cyclization.

**Scheme 16:**
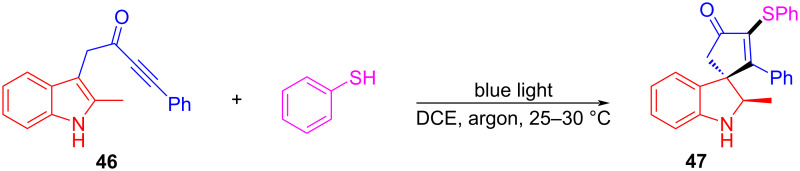
Synthesis of indoline derivative **47** initiated by an EDA complex.

**Scheme 17:**
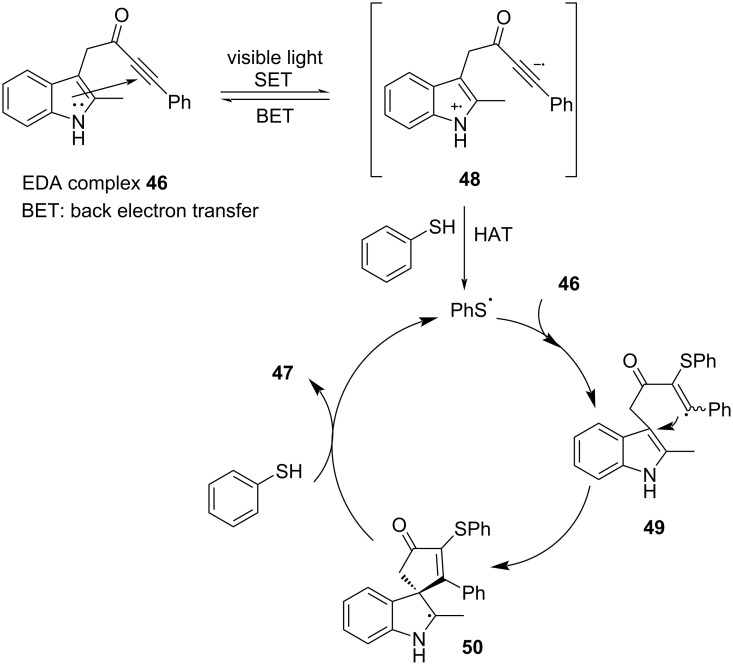
Mechanism of the synthesis of spirocyclic indoline derivative **47**.

In 2020, Taylor and colleagues [26] proposed a reaction for the preparation of spirocyclic indoline derivative **47** from indolylynone **46** and thiophenol under blue-light irradiation (Scheme 16). An abundant range of products was given to test various indole-tethered ynones and thiols, confirming that the reaction is broad in scope. Remarkably, C–S bonds and spiro compounds have been constructed simultaneously in this approach, which are promising for drug synthesis. Substrate **46** comprises both indole and alkynone groups, leading to light-promoted intramolecular electron transfer in order to form radical intermediate **48**. Then, **48** absorbs the hydrogen atom of thiophenol, yielding a thiophenol radical. The addition of the thiophenol radical to alkynone forms radical intermediate **49**. Radical intermediate **50** is given due to the cyclization of radical intermediate **49**, followed by abstracting hydrogen atoms from thiophenol to produce the final product **47** (Scheme 17).

**Scheme 18:**
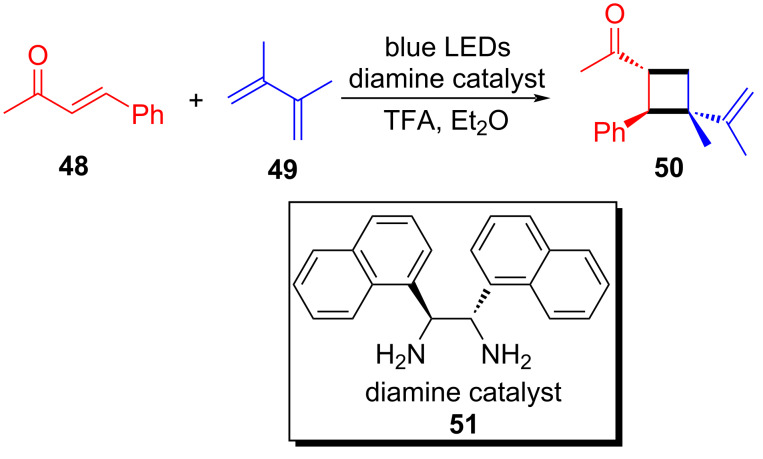
Synthesis of cyclobutane product **50** initiated by an EDA complex.

In 2020, Alemán and colleagues [53] proposed an approach in which ketene **48** and diene **49** condense with the help of diamine **51** to afford cyclobutane product **50** (Scheme 18). The reaction could be catalyzed by a simple diamine due to the fact that diamine can condense with the enol substrate, forming an imine-ion intermediate absorbing in the visible region. The direct excitation of the intermediate leads to a charge-transfer excited state, completing the stereocontrolled intermolecular cycloaddition reaction with a good ratio of enantiomer to diastereomer.

**Scheme 19:**
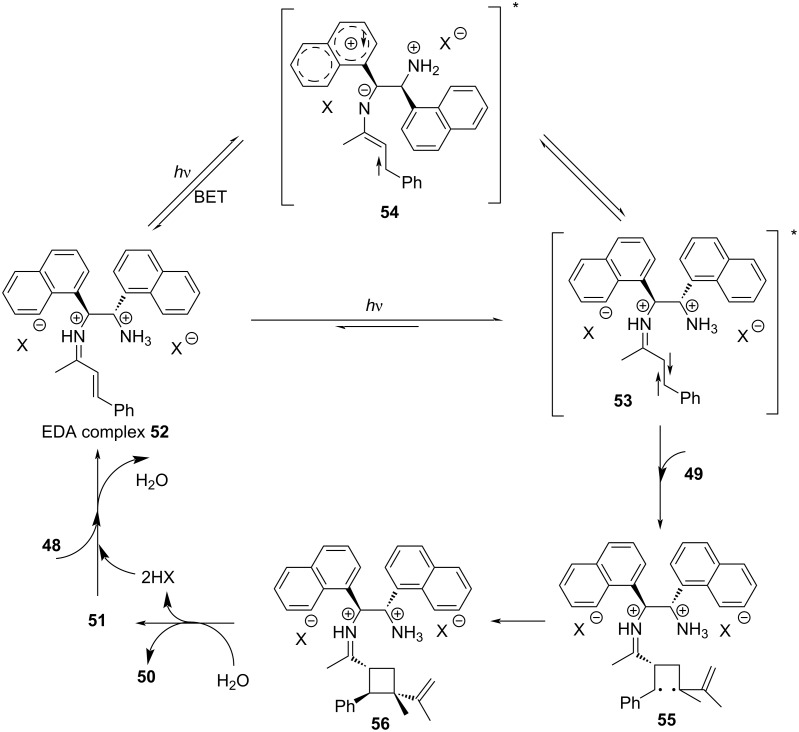
Mechanism of the synthesis of spirocyclic indoline derivative **50**.

A plausible mechanism is shown in Scheme 19. In the presence of acid, EDA complex **52** is formed by ketene **48** and diamine **51**. Then, the ground state **52** transforms into excited state **53** or into unproductive charge-transfer excited state **54** that can restore ground state **52** by BET; moreover, **53** and **54** can be mutually transformed. Excited state **53** reacts with diene **49**, forming a double radical intermediate **55** that subsequently evolves to cyclobutyliminium ion **56**, and then product **50** is provided after hydrolysis, along with the release of diamine **51**.

**Scheme 20:**
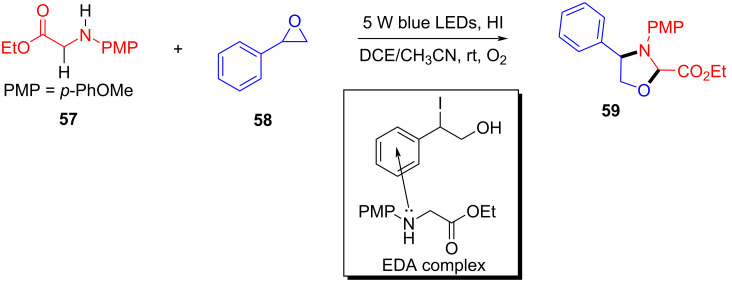
Synthesis of 1,3-oxazolidine compound **59** initiated by an EDA complex.

In 2020, Zhang and colleagues [25] developed a visible-light-induced [3 + 2] cycloaddition reaction between glycine derivatives **57** and aryl epoxides **58**, which can efficiently prepare a series of multisubstituted 1,3-oxazolidines **59** at room temperature (Scheme 20). The strategy can be applied smoothly to an extensive range of glycine derivatives, including electron-donating or electron-withdrawing substituent groups in the para- or meta positions at the benzene rings, giving corresponding products in moderate yield. This protocol is also suitable for the structural diversity of epoxides, providing a new activation approach for C(sp^3^)–H-functionalization of glycine derivatives.

### The construction of C–C bonds

As a crucial element in the construction of various organic scaffolds, the formation of C–C bonds remains a hot topic in the field of synthetic organic chemistry. The conventional approaches of C–C-bond construction typically employ transition-metal catalysts, such as in the Suzuki–Miyaura and Heck coupling reactions. Methodologies for forming different C–C bonds have recently been developed in the field of single-electron chemistry [63–65]. Considering that EDA-complex-initiated free-radical reactions are carried out under mild conditions, more attention has been paid to this efficient strategy for C–C-bond formation.

**Scheme 21:**
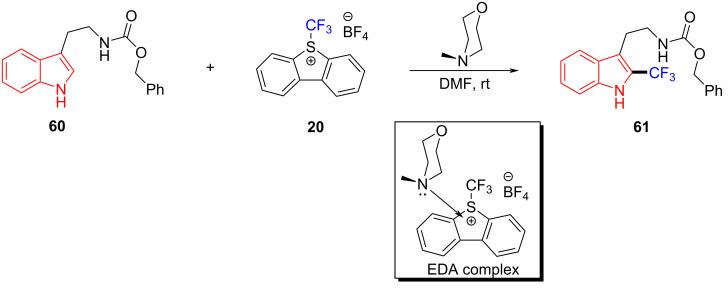
Synthesis of trifluoromethylated product **61** initiated by an EDA complex.

In 2015, Yu and colleagues [27] proposed a method for direct C–H trifluoromethylation of aromatic hydrocarbons through an EDA complex. Trifluoromethylated product **61** was synthesized by employing tryptamine derivative **60** and Umemoto reagent **20** as substrates as well as *N*-methylmorpholine (NMM) as organic base additive at room temperature (Scheme 21). The highly functionalized indole, pyrrole, benzofuran, and electron-rich benzene containing CF_3_ can be given in good yield. Given the redox potential of NMM and Umemoto reagent, directly conducting thermodynamic intermolecular SET is impossible. Thus, it is worth noting that the SET in this approach can be carried out only at room temperature without visible light.

**Scheme 22:**
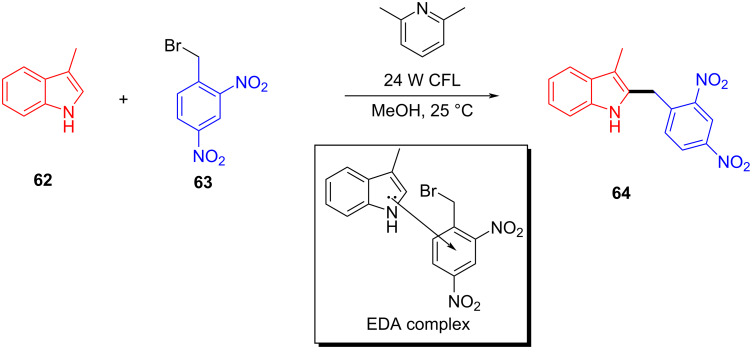
Synthesis of indole alkylation product **64** initiated by an EDA complex.

In 2015, Melchiorre and colleagues [28] proposed a visible-light-induced reaction to synthesize indole alkylation product **64** by exploiting the EDA complex formed by 3-methylindole (**62**) and 2,4-dinitrobenzyl bromide (**63**), with 2,6-dimethylpyridine as additive at room temperature (Scheme 22). The substrates with different substituents at position C2 and C3 of indole have been synthesized smoothly, including *cis*-fused pyrrolo- and furanoindolines. The X-ray single-crystal analysis showed that the EDA complex is received in the form of a face-to-face π–π complex, and the ratio of donor to acceptor is 1:1.

**Scheme 23:**
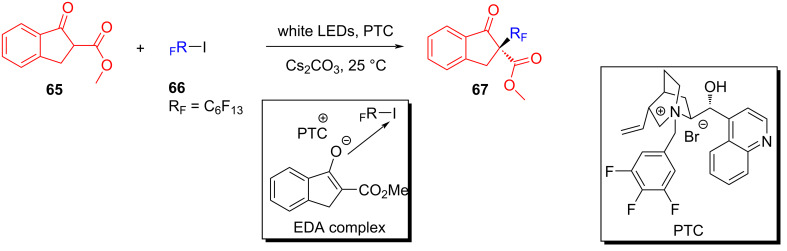
Synthesis of perfluoroalkylation product **67** initiated by an EDA complex.

In the same year, based on previous experimental phenomena and data, Melchiorre and colleagues [45] designed a reaction, with indanone derivatives **65** and perfluorohexyl iodide (**66**) as substrates and a phase-transfer catalyst (PTC) to give perfluoroalkylation product **67** under white-light irradiation (Scheme 23). A variety of electron-withdrawing substituents on the aromatic ring of **65** were well tolerated; however, the presence of electron-donating substituents lowered the reactivity due to a negative impact on the EDA complex formation and led to a low yield. It is worth noting that the phase-transfer catalyst employed in this experiment is a suitable donor for the photosensitive EDA complex while at the same time providing effective asymmetric induction in the capture of the resulting radical along with enantioselectivity of the product.

**Scheme 24:**
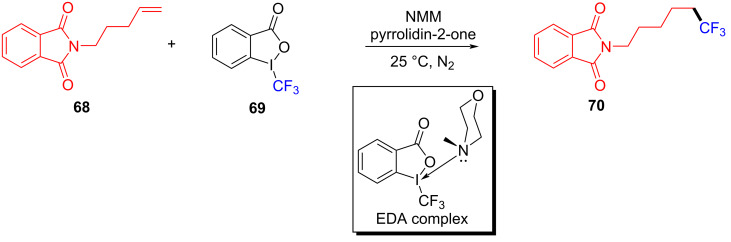
Synthesis of hydrotrifluoromethylated product **70** initiated by an EDA complex.

In 2016, based on the experimental work in 2015 [27], Yu and colleagues [29] reported a type of EDA complex that could complete the hydrotrifluoromethylation of unactivated olefins and alkynes. This approach employed **68** and Togni reagent **69** (electron acceptor) as substrates, NMM as electron donor, and pyrrolidin-2-one as solvent to give hydrotrifluoromethylated product **70** at room temperature (Scheme 24). CF_3_ was added to a variety of terminal alkenes, leading to corresponding hydrotrifluoromethylation products with moderate to good yield.

**Scheme 25:**
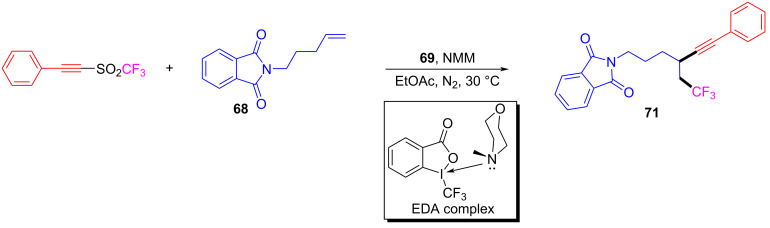
Synthesis of β-trifluoromethylated alkyne product **71** initiated by an EDA complex.

In 2017, Yu and colleagues [30] proposed an EDA-complex-induced alkyne trifluoromethylation reaction. The EDA complex formed by a catalytic quantity of Togni reagent **69** and NMM initiated the chain propagation, causing the final alkyne trifluoromethylation (Scheme 25). A variety of olefins, such as ene carbamates, styrene, aliphatic olefins, vinyl ethers, and acrylates are compatible in this approach, affording corresponding β-(trifluoromethyl)alkynes with good to excellent yield. The bifunctionalization was achieved by an EDA-complex-initiated three-component reaction in the absence of light, which is of great significance for the later study of temperature-driven EDA complexes.

**Scheme 26:**
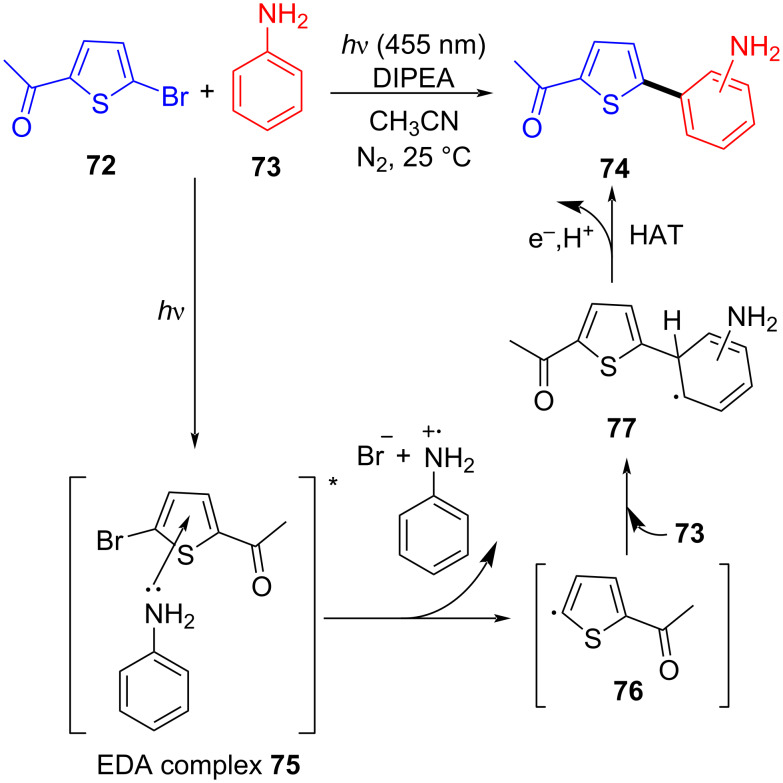
Mechanism of the synthesis of 2-phenylthiophene derivative **74**.

In 2017, König and colleagues [42] discovered an EDA complex **75** formed by bromothiophene **72**, aniline (**73**), and *N*,*N*-diisopropylethylamine (DIPEA) as organic base additive to give corresponding thiophene radical **76** and aniline radical cation under irradiation with light. Then, **76** reacted with **73**, giving rise to corresponding radical **77**. Finally, product **74** was given via hydrogen atom transfer (Scheme 26). In contrast to (hetero)aryl halides with indispensable electron-withdrawing groups, the scope of the reaction comprises anilines including electron-withdrawing or electron-donating substituents in the arene, except *N*-acetylated or ortho-halogenated anilines.

**Scheme 27:**
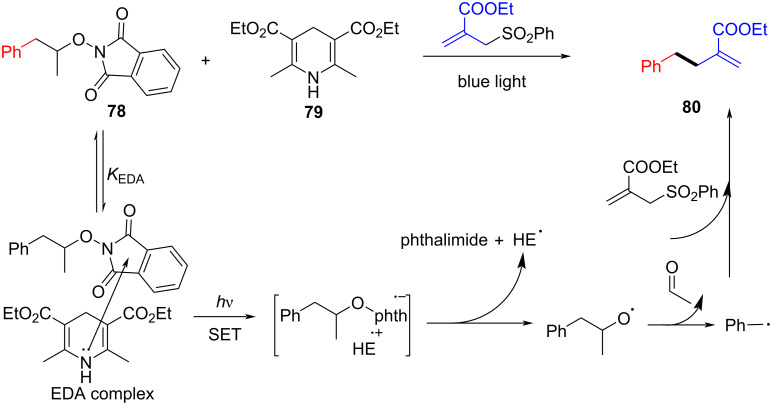
Synthesis of allylated product **80** initiated by an EDA complex.

In 2017, Chen and colleagues [21] reported a method that promoted the formation of an alkoxy radical through an EDA complex under visible-light irradiation. The EDA complex formed by electron donor Hantzsch ester (HE) **79** and electron acceptor *N*-acyloxybenzamide **78** was produced by light-promoted SET, providing alkoxy radicals that could give carbon radicals by removing one molecule of acetaldehyde (Scheme 27). It is worth noting that EDA complex has been firstly employed for the generation of alkoxy radicals under visible-light irradiation, achieving selective C(sp^3^)–C(sp^3^)-bond cleavage and allylation or alkenylation.

**Scheme 28:**
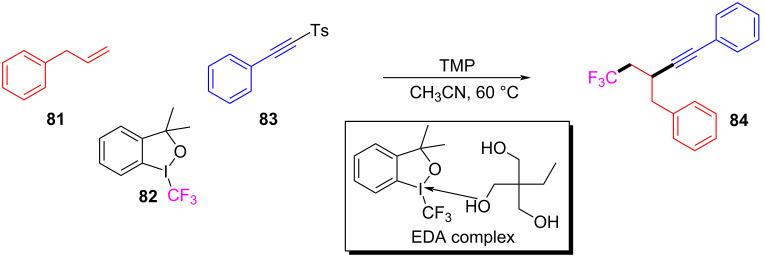
Synthesis of trifluoromethyl-substituted alkynyl product **84** initiated by an EDA complex.

In 2017, Li and colleagues [57] reported a reaction for the synthesis of trifluoromethyl alkynylation product **84** with alkynyl sulfone **83**, alkene **81**, and Togni reagent **82** as substrates catalyzed by 2,4,6-trimethylpyridine (TMP). The EDA complex formed by electron donor TMP and electron acceptor Togni reagent **82** facilitated electron transfer, yielding trifluoromethyl radical to initiate the subsequent reaction (Scheme 28). On account of a wide range of substrates and functional group compatibility, this protocol can be exploited to assemble various β-trifluoromethylated alkynes by three-component reaction without transition-metal catalysis.

**Scheme 29:**
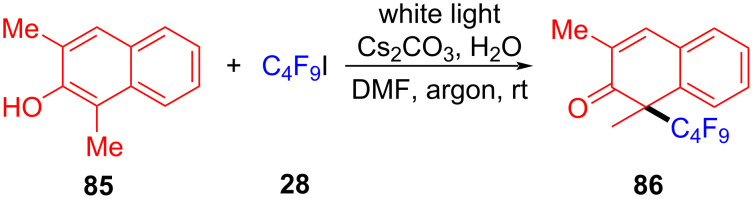
Synthesis of dearomatized fluoroalkylation product **86** initiated by an EDA complex.

**Scheme 30:**
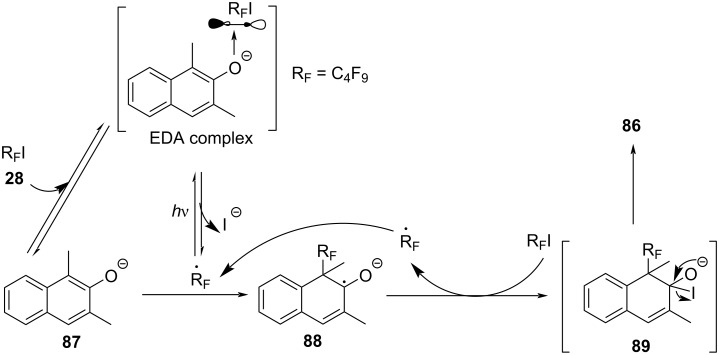
Mechanism of the synthesis of dearomatized fluoroalkylation product **86**.

In 2018, Xu and colleagues [48] proposed the dearomatization of β-naphthol promoted by visible light via intermolecular charge transfer (Scheme 29). In this method, β-naphthol anion **87** (β-naphthol **85** formed in the presence of base) is employed as electron donor to form EDA complex with electron acceptor perfluoroalkyl iodide **28**. Single-electron transfer occurs under white-light irradiation, leading to an electron-deficient fluoroalkyl radical. Thereafter, fluoroalkyl radical is captured by **87**, affording radical **88** that gives rise to radical intermediate **89** by uptake of iodine. Finally, the dealkylation product **86** is given by removing iodide anion (Scheme 30). Various β-naphthols with different substituents in the 1- and 3-positions were tolerated, providing the corresponding products with excellent yield.

**Scheme 31:**
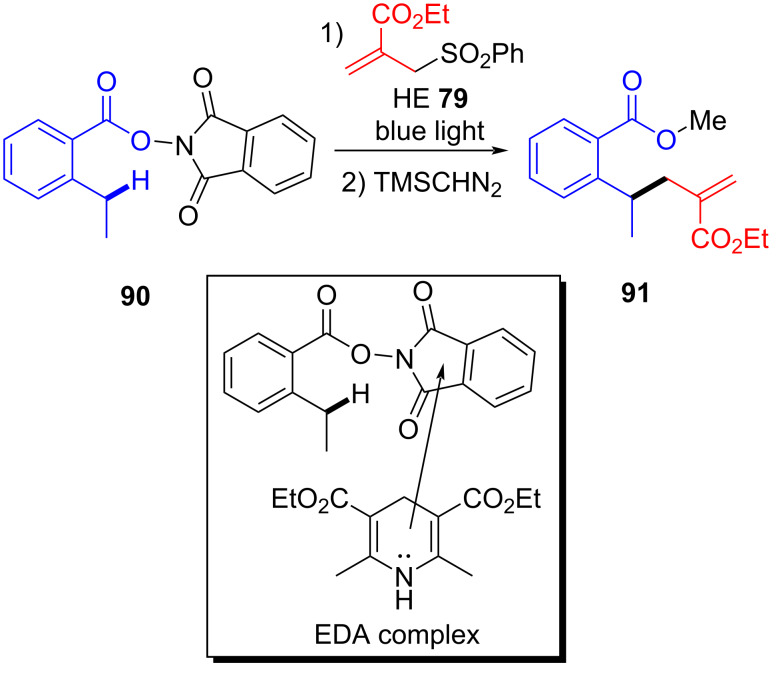
Synthesis of C(sp^3^)–H allylation product **91** initiated by an EDA complex.

In 2018, Chen and colleagues [18] reported a method to realize C(sp^3^)–H allylation by generating aryl carboxyl radical from EDA complex based on previous work in 2017 [21]. The reaction is initiated by the formation of EDA complex between electron acceptor *N*-acyloxyphthalimide **90** and electron donor HE **79**, completing regio- and chemoselective C(sp^3^)–H allylation or olefin bifunctionalization (Scheme 31).

**Scheme 32:**
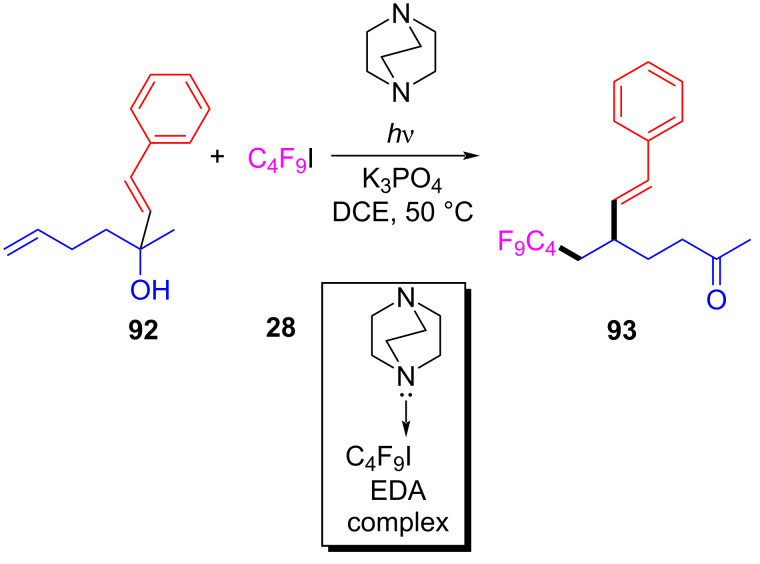
Synthesis of perfluoroalkylation product **93** initiated by an EDA complex.

In 2018, Tang and Studer [32] found a bifunctional group reaction of perfluoroalkylation and β-alkenylation by a perfluoroalkyl radical. Perfluoroalkylation product **93** was synthesized by utilizing (*E*)-3-methyl-1-phenylhept-1,6-dien-3-ol (**92**) and perfluorobutyl iodide (**28**) as substrates and potassium phosphate and DABCO as additives at 50 °C and under irradiation with light (Scheme 32). The reaction is compatible with phenyl substituents with high steric hindrance, indicating that steric effects of the aryl moiety in the migrating styrenyl group do not play a major role.

**Scheme 33:**
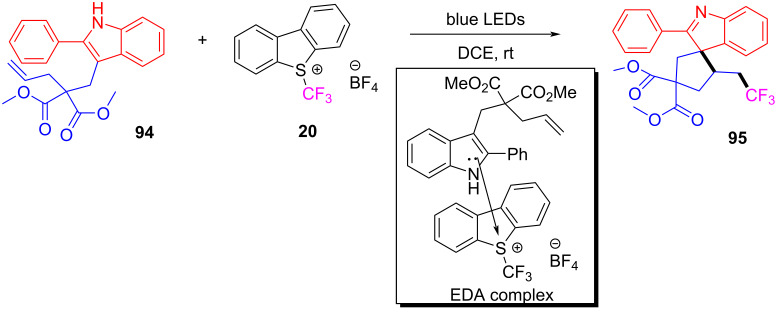
Synthesis of spirocyclic indolene derivative **95** initiated by an EDA complex.

In 2018, You and colleagues [19] reported the discovery of an EDA complex formed by indole derivative **94** and Umemoto reagent **20**, which provided the trifluoromethyl-substituted spirocyclic indolene **95** with stereoisomeric center in good yield (up to 90%) under blue-light irradiation (Scheme 33). A variety of groups have been tolerated at the C2 position of indole, including phenyl groups with electron-donating or electron-withdrawing substituents, as well as the alkyl moiety. To further investigate the practicability of this approach, **95** was synthesized smoothly on the gram scale with a yield of 70%.

**Scheme 34:**
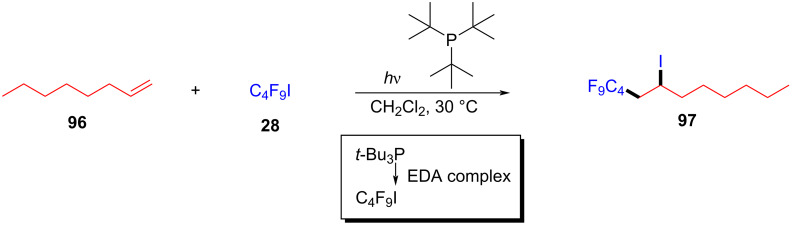
Synthesis of perfluoroalkylation product **97** initiated by an EDA complex.

In 2019, Czekelius and colleagues [43] found that the perfluoroalkylation of unactivated olefins can be realized with phosphine catalyst and perfluorobutyl iodide (**28**) under visible-light irradiation. The EDA complex formed by perfluorobutyl iodide (**28**) and phosphine catalyst induced SET, affording a perfluoroalkyl radical, and then perfluoroalkylation product **97** was yielded by addition of perfluoroalkyl radical with olefin **96** (Scheme 34). Upon the termination of the reaction, the desired product can be given by removing the solvent and precipitating the catalyst. The comparison experiments and electronic absorption spectra showed that the efficiency of the catalyst was related to enhancement of selective absorption, considering the use of a visible-light source.

**Scheme 35:**
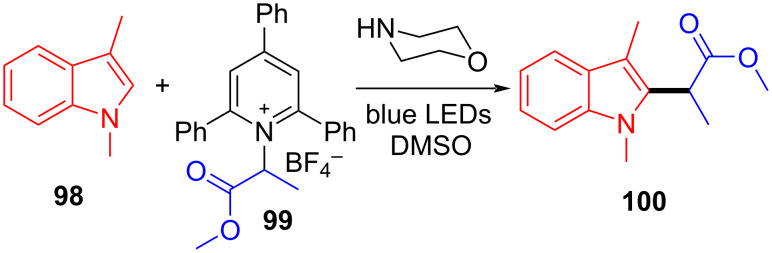
Synthesis of alkylated indole derivative **100** initiated by an EDA complex.

**Scheme 36:**
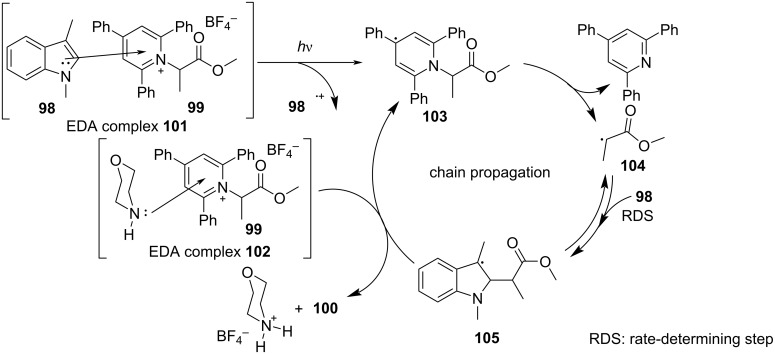
Mechanism of the synthesis of alkylated indole derivative **100**.

In 2019, Glorius and colleagues [11] proposed a method of employing an EDA complex formed by indole derivative **98** and the Katritzky salt **99** as well as morpholine as organic base to obtain alkylated indole derivatives **100** under blue-light irradiation (Scheme 35). Given the UV–vis spectra of the reaction mixture and its components, there was no evidence of the formation of a ternary EDA complex between Katritzky salt **99**, **98**, and morpholine. Moreover, the TDDFT calculation showed that electron transfer took place in complex **101** under visible-light excitation. Hence, it can be inferred that radical-chain propagation was initiated by a small amount of radicals that emerged from excited complex **101**. The C–N bond in dissociated radical **103** is irreversibly broken, along with the appearance of radical **104**. Subsequently, alkyl radical **104** is captured by indole **98**, giving benzyl radical **105**. The alkylated indole derivative **100** and morpholine salts are provided via proton-coupled electron transfer (PCET) with EDA complex **102** formed by morpholine and **99** (Scheme 36). As a rare example of EDA photochemistry, two kinds of EDA complexes were involved in this approach, explaining the reason why the yield increased significantly when morpholine was employed as an organic base additive, which was exploited in the screening stage of the reaction conditions.

**Scheme 37:**
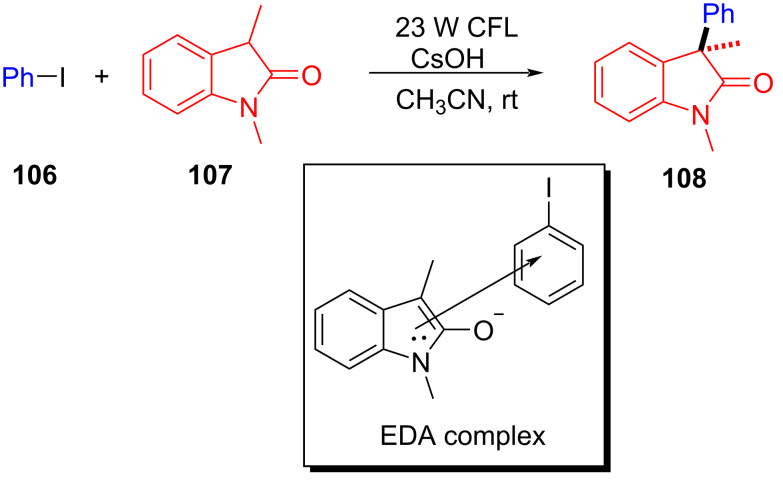
Synthesis of arylated oxidized indole derivative **108** initiated by an EDA complex.

In 2019, Xia and colleagues [46] reported that the EDA complex formed by aryl halide **106** and oxindole **107** under alkaline conditions allowed single-electron transfer under irradiation with light, eventually affording arylated oxidized indole product **108** (Scheme 37). This reaction provides an effective method to construct various 3-arylindoles with medicinal value at ambient temperature, which has a wide range of substrates, including various (hetero)aryl halides and substituted oxindoles.

**Scheme 38:**
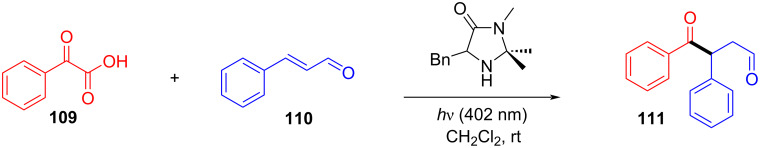
Synthesis of 4-ketoaldehyde derivative **111** initiated by an EDA complex.

**Scheme 39:**
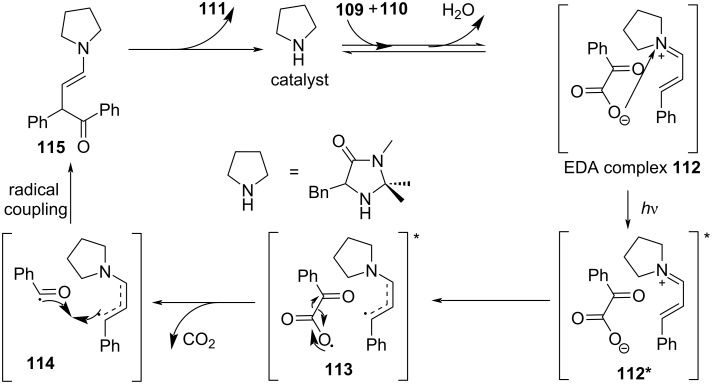
Mechanism of the synthesis of 4-ketoaldehyde derivative **111**.

In 2019, Gilmour and colleagues [12] transformed the classical Stetter reaction into a radical approach, solving the long-standing problem of chemical selectivity to convert α,β-unsaturated aldehydes selectively into 4-ketoaldehydes (Scheme 38). The imine salt (electron acceptor) that forms EDA complex **112** with electron donor α-keto acid **109** is synthesized by secondary amine catalyst and α,β-unsaturated aldehyde **110**. Compound **112** turns to excited state 112* under irradiation with light. Then, radical intermediate **113** is afforded via intermolecular electron transfer, followed by removing one molecule of carbon dioxide to give radical intermediate **114**. Species **115** is formed through radical coupling in **114**, providing the target product **111** with the release of the secondary amine catalyst (Scheme 39).

**Scheme 40:**
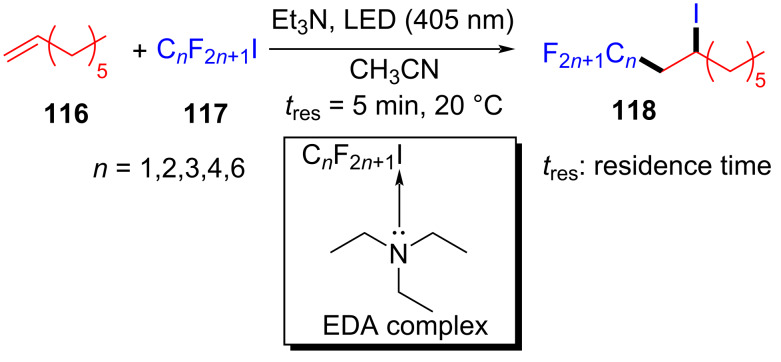
Synthesis of perfluoroalkylated olefin **118** initiated by an EDA complex.

In 2019, Kappe and colleagues [36] reported a method to complete perfluoroalkylation of olefins under visible light in flow. Perfluoroalkylated olefin **118** was prepared by employing olefin **116** and perfluoroalkyl iodides **117** as substrates as well as triethylamine as additive at 20 °C and under 405 nm irradiation (Scheme 40). A standard residence time of 5 min was required for the full conversion via the EDA complex that formed by alkene and perfluoroalkyl iodide in flow, while longer residence times were requisite for less reactive alkenes. Moreover, the yield of this reaction can reach 7.6 g ⋅ h^−1^ on a gram scale, indicating that the flow step is promising in photochemistry.

**Scheme 41:**
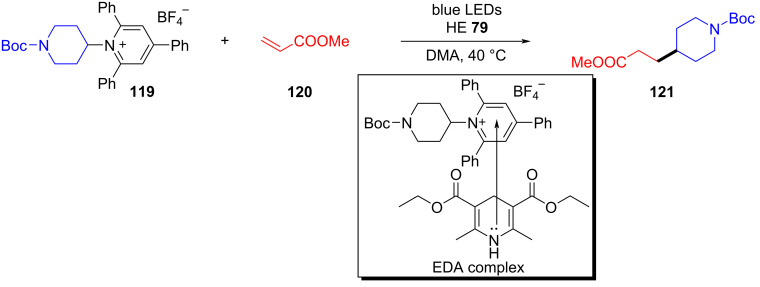
Synthesis of alkylation product **121** initiated by an EDA complex.

In 2019, Aggarwal and colleagues [38] employed Katritzky salt **119** as electron acceptor and HE **79** as electron donor to form an EDA complex, providing the corresponding alkyl radical that could react with olefin **120** with an electron-withdrawing group to give alkylation product **121** under irradiation with light (Scheme 41). The reaction is compatible with various substrates, including alkenes, secondary alkylpyridinium ions, benzylic pyridinium ions, and primary alkylpyridinium ions, which can be considered an effective method for the generation of alkyl radicals without catalyst.

**Scheme 42:**
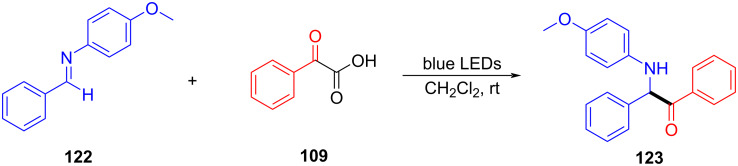
Synthesis of acylation product **123** initiated by an EDA complex.

**Scheme 43:**
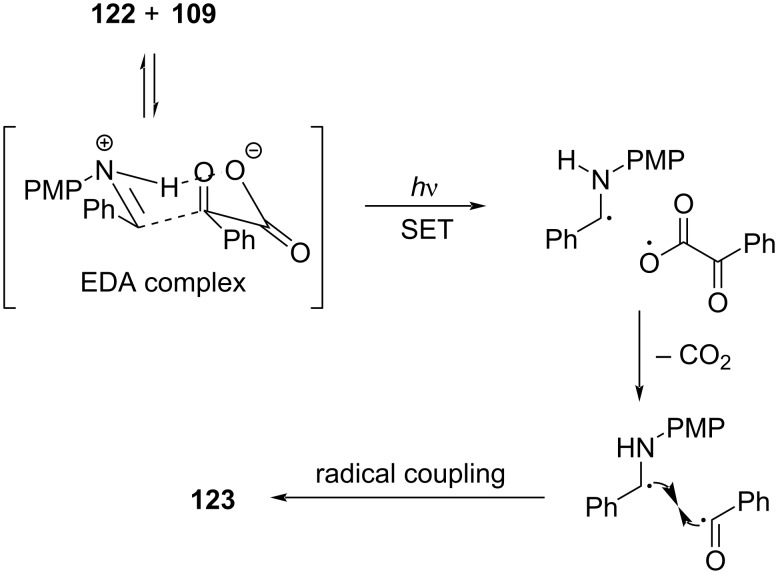
Mechanism of the synthesis of acylation product **123**.

In 2019, Yu and Zhang [15] reported a radical acylation reaction initiated by an EDA complex promoted by visible light. Imine **122** was employed as electron acceptor with α-keto acid **109** as electron donor to form the EDA complex, affording acylation product **123** under blue-light irradiation (Scheme 42). The quantum yield of the reaction was determined to be 0.08, suggesting that the reaction proceeded via radical coupling rather than a radical propagation. Moreover, the reaction was compatible with amides, cyanides, esters, ethers, halides, and heterocycles, and various α-aminoketones (32 examples) can be yielded in 90% isolated yield. According to the author, the EDA complex had a six-membered-ring transient state, and the imine also acted as an organic base (abstracting proton from α-keto acid), proving that electron transfer is accompanied by proton transfer in the process (Scheme 43).

**Scheme 44:**
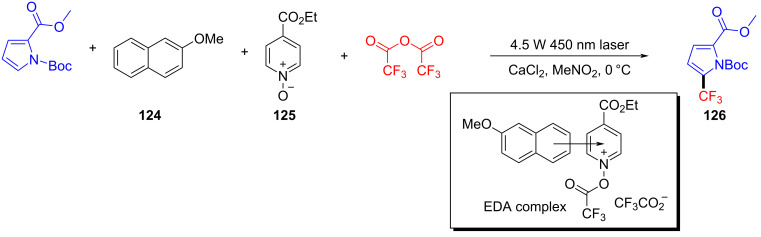
Synthesis of trifluoromethylation product **126** initiated by an EDA complex.

In 2020, Stephenson and colleagues [14] employed 2-methoxynaphthalene (**124**) and acylated ethyl isonicotinate *N*-oxide obtained from **125** and trifluoroacetic acid anhydride to form EDA complexes for the preparation of trifluoromethylated product **126** (Scheme 44). As a rare example of EDA photochemistry in the catalytic system, only a catalytic equivalent of the electron donor was employed in this approach. Further experiments showed that the addition of inorganic salts, calcium chloride and lithium chloride, could increase the absorption of EDA complex in the visible-light region.

**Scheme 45:**
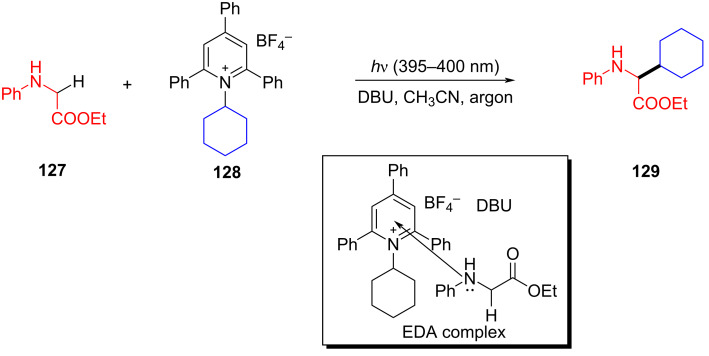
Synthesis of unnatural α-amino acid **129** initiated by an EDA complex.

In 2020, Xu and colleagues [39] proposed a visible-light-promoted alkylation reaction using Katritzky salts such as **128** and glycine derivative **127** (or glycine segments in peptides) initiated by an EDA complex. This successfully realized the simple synthesis of unnatural α-amino acids **129** and precise alkylation modification of peptides in the later stage (Scheme 45). Even in the presence of other amino acid residues, this protocol has excellent regio- and chemoselectivity, providing a sequence of novel corresponding dipeptides with good yield.

### The construction of C–S bonds

C–S bonds are commonly present in amino acids, proteins, glycosides, nucleic acids, and other biological macromolecules. In recent years, photocatalyst- and transition-metal strategies have been employed to construct C–S bonds [66–69]. The C–S bond synthesis via EDA-complex pathways has the advantages of mild reaction conditions and a high tolerance to functional groups, which can be exploited for artificial syntheses of biological macromolecules.

**Scheme 46:**
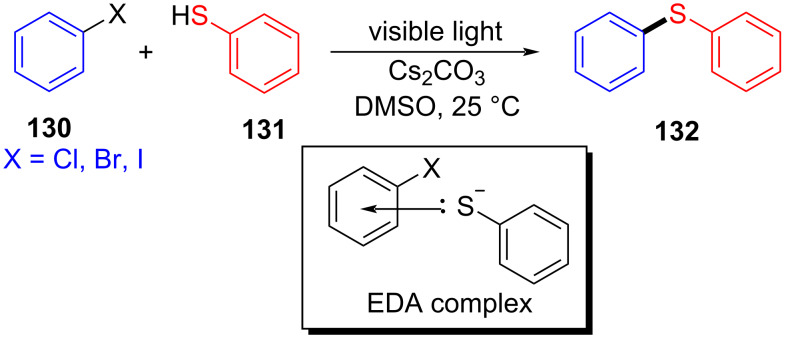
Synthesis of thioether derivative **132** initiated by an EDA complex.

In 2017, Miyake and colleagues [54] designed a type of C–S cross-coupling reaction initiated by an EDA complex promoted by visible light. In this approach, halogenated aromatic **130** was employed as electron acceptor with thiophenol (**131**) as electron donor to form an EDA complex. Light-promoted intermolecular electron transfer took place to give corresponding radicals, respectively, in the presence of base, and then cross-coupling between radicals was carried out, affording thioether derivative **132** (Scheme 46). It has been proved by UV–vis spectroscopy and TDDFT calculations that the EDA complex was formed between an electron-rich mercaptan anion and electron-deficient aryl halides. Most importantly, this approach can be successfully applied to the gram scale, providing a step towards assorted aryl sulfide structural units with medicinal value.

**Scheme 47:**
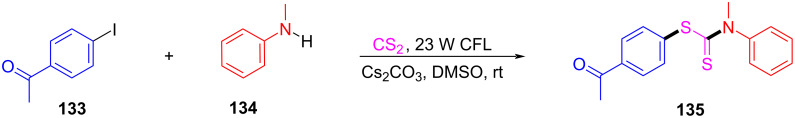
Synthesis of *S*-aryl dithiocarbamate product **135** initiated by an EDA complex.

In 2019, Yang and colleagues [52] developed a method for preparing *S*-aryl dithiocarbamates **135** by a multicomponent reaction of an EDA complex under visible-light irradiation (Scheme 47). A number of aryl halides reacted smoothly, providing moderate to good yields for analogous *S*-aryl dithiocarbamates. To further demonstrate the synthetic application of this protocol, a gram scale of **135** has been tested, giving a yield of 72%. By constructing C–N- and C–S bonds simultaneously in one step without any transition-metal catalyst, ligand, or photocatalyst, this method possesses a splendid application prospect.

**Scheme 48:**
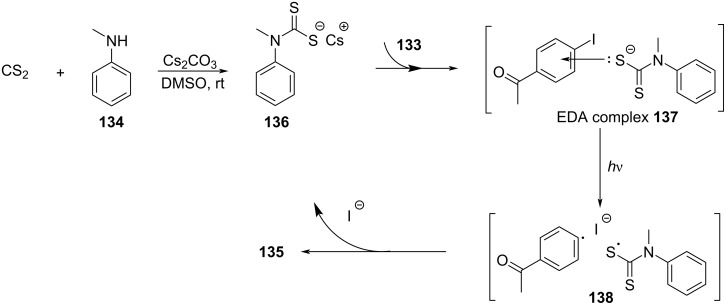
Mechanism of the synthesis of *S*-aryl dithiocarbamate product **135**.

The reaction mechanism is as follows (Scheme 48): Firstly, carbon disulfide combines with *N*-methylaniline (**134**) in the presence of Cs_2_CO_3_ to form thiolate **136**. Thiolate **136** is employed as electron donor to generate EDA complex **137** with halogenated electron-acceptor aromatics **133**, and then electron transfer occurs, affording intermediate **138**. Finally, radical coupling gives rise to *S*-aryl dithiocarbamate product **135**.

**Scheme 49:**
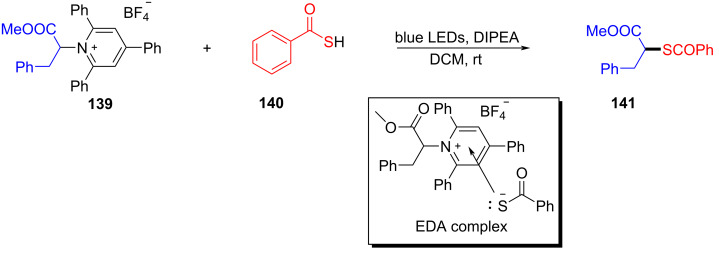
Synthesis of thioether product **141** initiated by an EDA complex.

In 2019, Liao and colleagues [13] utilized Katritzky salt **139** and thiobenzoic acid (**140**) to form an EDA complex, providing thioether derivative **141** with DIPEA as an organic base additive (Scheme 49). This reaction offers a novel and simple approach for the synthesis of α-mercapto acid derivatives under mild reaction conditions and demonstrates strong compatibility to the functional group. In addition, a gram-scale reaction also gives the desired thioether product in a yield of 99%.

### The construction of C–B bonds

The C–B bond can be converted into a wide range of other functional groups by the conversion of alkyl borane [70–72]. Hence, it has become imperative to pursue more efficient syntheses for constructing C–B bonds. In recent years, the construction of C–B bonds via EDA complexes has attracted more chemists' attention.

**Scheme 50:**
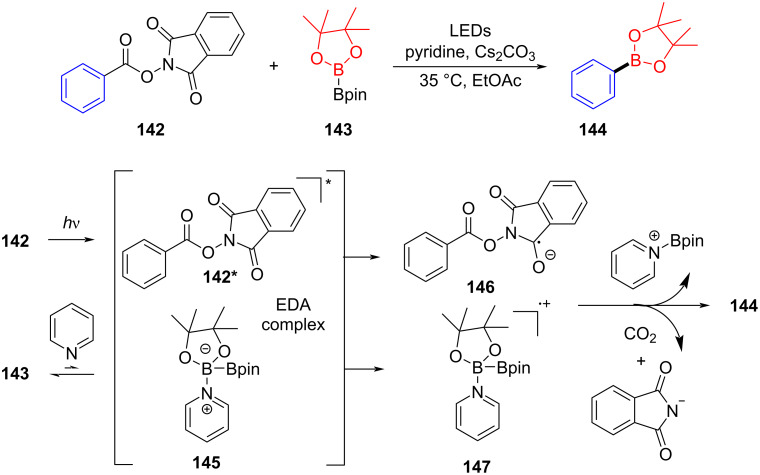
Mechanism of the synthesis of borate product **144**.

In 2017, Glorius and colleagues [51] discovered a visible-light-induced decarboxylated borate of arylcarboxylic acid initiated by an EDA complex. First, the *N*-hydroxyphthalimide (NHPI) ester **142** is excited to electron acceptor 142* through visible-light intersystem crossing (ISC); diborate **143** combining with pyridine results in electron donor **145**. Upon the formation of the EDA complex between **145** and 142*, electron transfer occurs, giving radical **146** and radical cation **147**, respectively. Finally, radical **146** undergoes decarboxylation to afford an aryl radical and then combines with radical cation **147**, yielding product **144** (Scheme 50). It should be noted that only when NHPI is firstly activated can it turn into an electron acceptor, and thus further combines with the electron donor to form an EDA complex, mainly due to the fact that the electron acceptor in an excited state allows for stronger oxidation, to integrate with the electron donor.

**Scheme 51:**
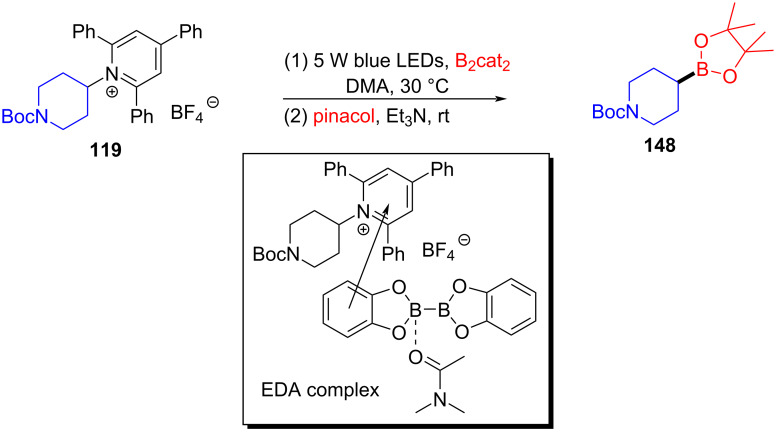
Synthesis of boronation product **148** initiated by an EDA complex.

In 2018, Glorius and colleagues [16] reported a method for the preparation of boron-substituted product **148** by employing Katritzky salt **119** as electron acceptor as well as the complex formed by bis(catecholato)diboron (B_2_cat_2_) and solvent DMA as electron donor to afford an EDA complex (Scheme 51). This approach can effectively convert primary-, benzyl-, and secondary amines into corresponding borated products, with only a coordinating solvent, DMA. Furthermore, functionalization of natural products and drug molecules has been accomplished smoothly with excellent yields.

**Scheme 52:**
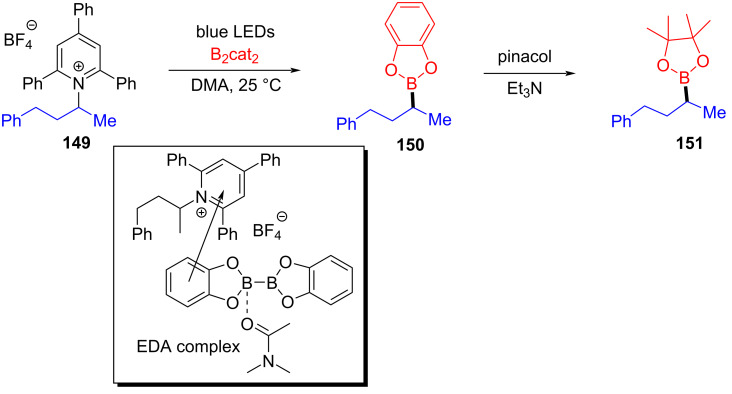
Synthesis of boration product **151** initiated by an EDA complex.

In 2018, Aggarwal and colleagues [17] proposed a method for preparing alkyl borate derivative **150** by employing Katritzky alkylpyridinium salt **149** and B_2_cat_2_ as substrates as well as DMA as coordination solvent under blue-light irradiation, followed by subsequent reaction with pinacol to afford boration product **151** (Scheme 52). A number of secondary alkylamines, even those that have carbamate- or phthalimide-protected amines, have been efficiently transformed to suitable pinacol boronic esters. This simple operation without transition-metal catalysis will be widely promoted in the synthesis of important boron-containing molecules in medicine and biology.

**Scheme 53:**
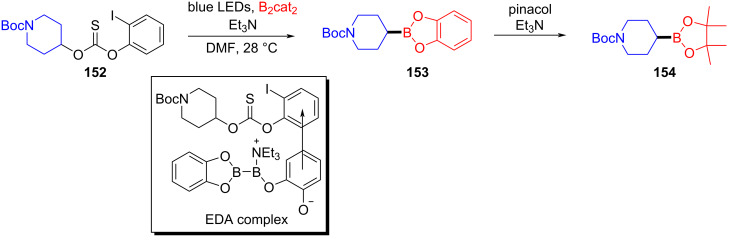
Synthesis of boronic acid ester derivative **154** initiated by an EDA complex.

In 2019, Aggarwal and colleagues [55] proposed that the EDA complex was formed by 2-iodophenyl thiocarbonate **152**, bis(catecholato)diboron, and triethylamine, which afforded boronic acid ester derivative **153** under blue-light irradiation. Simultaneously, pinacol boronic acid ester derivative **154** can be yielded by subsequent processing (Scheme 53). The protocol reveals a high functional-group tolerance that permits the transformation into boronic esters of natural alcohol products with high stereocontrol.

### The construction of C–N bonds

The development of efficient methods to construct C–N bonds is an essential scheme in organic synthesis due to its widespread presence in pharmaceutical-, agrochemical-, and materials sciences [73–75]. At present, most of the C–N-bonding reactions require transition-metal catalysis, and the reaction conditions are more stringent; however, the EDA-complex pathway proceeds under mild, catalyst-free conditions, promoted by irradiation with visible light.

**Scheme 54:**
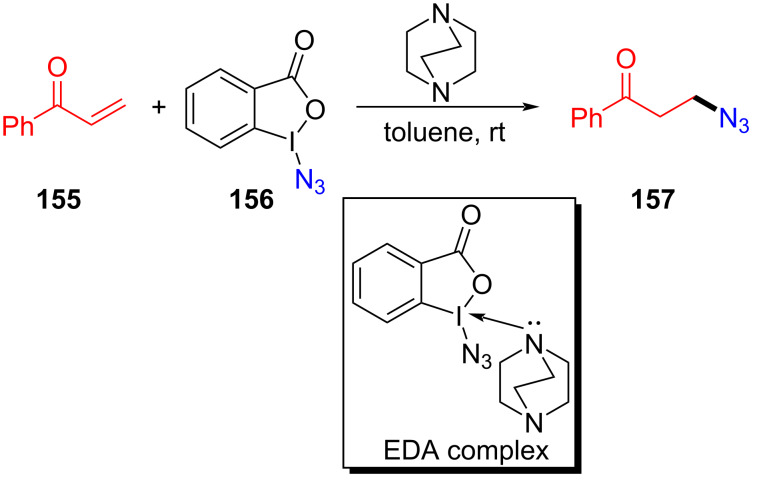
Synthesis of β-azide product **157** initiated by an EDA complex.

In 2017, Shirke and Ramaastry [40] proposed an organic catalyzed β-azide reaction of ketene **155** initiated by the EDA complex formed by DABCO and Zhdankin reagent **156** (Scheme 54). A variety of β-azidoketones was conveniently obtained with good to excellent yield with electron-rich as well as electron-poor arenes and heteroarenes. Subsequently, in order to prove the practicability of this approach, 1,2,3-triazoles were assembled by reaction of **157** with alkynes.

**Scheme 55:**
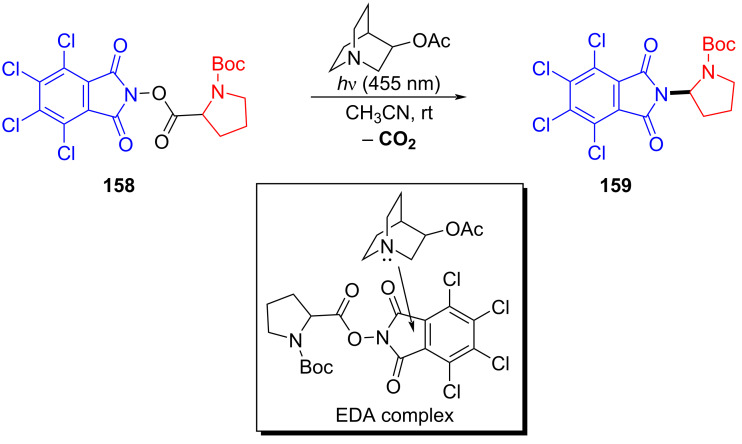
Decarboxylation reaction initiated by an EDA complex.

In 2019, Bosque and Bach [41] reported that 3-acetoxyquinuclidine (q-OAc) could be utilized as an electron-donor catalyst to form an EDA complex with electron acceptor **158**, and then a molecule of carbon dioxide was removed under 455 nm light irradiation, giving decarboxylation product **159** (Scheme 55). It was found that many ester groups can be activated by the structural motif of tetrachlorophthalimide in **158**. Significantly, in contrast to most traditional EDA complex approaches that consume the DA pair, the electron-donor catalyst q-OAc in this method could be regenerated.

**Scheme 56:**
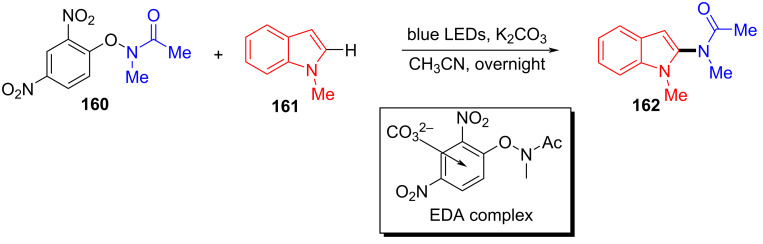
Synthesis of amidated product **162** initiated by an EDA complex.

In 2019, Frontera and colleagues [49] obtained target product **162** with blue LEDs irradiation of a solution containing electron-poor *N*-aryloxyamides **160**, indole derivative **161**, and carbonate or other multicharge anions in CH_3_CN (Scheme 56). The corresponding products can be given in good yield by modifying substituents on the amide moiety in **160** or *N*-substituted indoles. Inorganic-base electron donors formed transient complexes with *N*-aryloxyamides, driven by noncovalent anion–π interactions, which has been described for the first time in a light-promoted process.

### The construction of C–P bonds

Many compounds contain phosphorus, which has gained a high degree of interest in materials, agriculture, medical science, and biology [76]. Cases of C–P bond construction employing photoredox [77–78] or photoredox/nickel dual catalysis [79] have been identified in the field of photochemistry. However, here we introduce the methods initiated by EDA complexes for C–P bond construction.

**Scheme 57:**
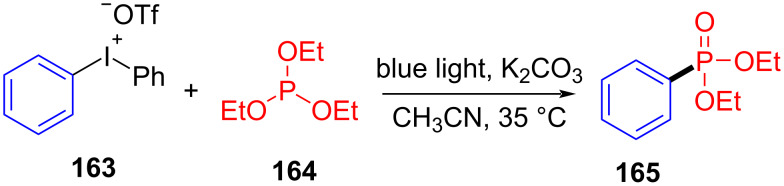
Synthesis of diethyl phenylphosphonate **165** initiated by an EDA complex.

**Scheme 58:**
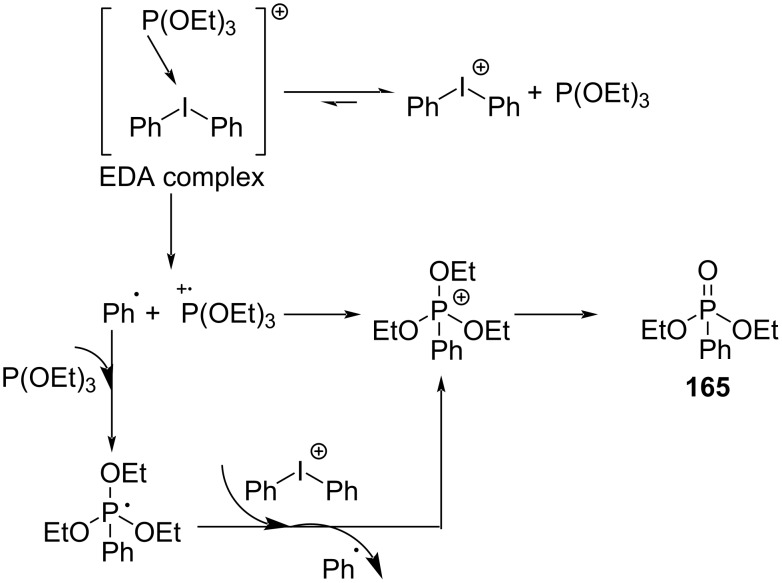
Mechanism of the synthesis of diethyl phenylphosphonate derivative **165**.

In 2018, Lakhdar and colleagues [44] reported a visible-light-mediated synthesis approach of arylphosphonates initiated by an EDA complex. Diethyl phenylphosphonate (**165**) was given by exploiting diphenyliodonium trifluoromethanesulfonate (**163**) as electron acceptor, triethylphosphite (**164**) as electron donor, potassium carbonate as base, and CH_3_CN as solvent (Scheme 57). The complex is bound together by weak halogen bonds, in which phosphorus lone-pair electrons interact with σ* orbitals of C–I bonds. A variety of arylphosphonates can be directly afforded by the simple combination of diaryliodonium salts and phosphite esters. In addition, calculations including EPR, NMR, and DFT have been carried out to prove that the reaction mechanism is consistent with inference (Scheme 58).

### The construction of C–O bonds and C–H bonds

Although there have been few cases of constructing C–O bonds and C–H bonds via EDA-complex pathways in recent years, we also summarized them in view of their great significance in organic synthesis.

**Scheme 59:**
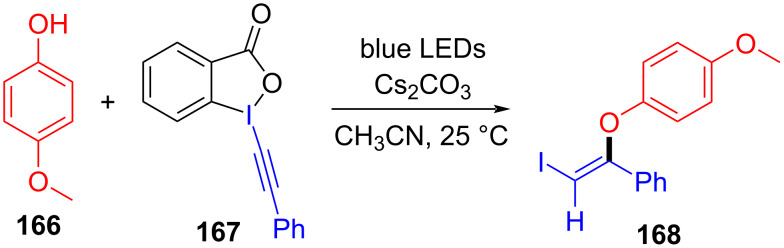
Synthesis of (*Z*)-2-iodovinyl phenyl ether **168** initiated by an EDA complex.

In 2018, Miyake and colleagues [50] found a protocol for the preparation of (*Z*)-2-iodovinyl phenyl ether **168** by utilizing ethynylbenziodoxol(on)e (EBX) **167** and phenol derivative **166** (Scheme 59). Due to the lack of significant electronic effects of phenol, a variety of phenols, including electron-donor and electron-withdrawing groups, were been converted into corresponding 2-iodovinyl phenyl ethers in moderate to excellent yield with high regio- and stereoselectivities.

**Scheme 60:**
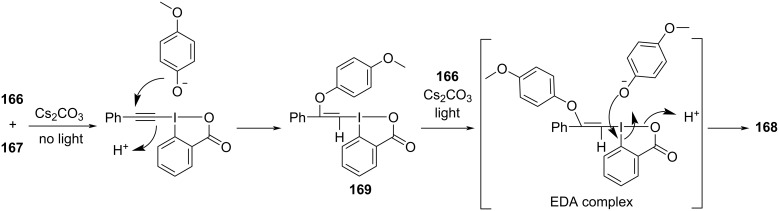
Mechanism of the synthesis of (*Z*)-2-iodovinyl phenyl ether derivative **168**.

According to the analysis of the mechanism (Scheme 60), a molecule of phenol anion is first added to the alkyne group of an EBX, forming electron acceptor **169**, which causes the destabilization of the C–I bond. Then, electron acceptor **169** forms an EDA complex with phenol anion, along with light-promoted electron transfer occurring. Thereby, the C–I bond and the I–O bond break to afford the final product (*Z*)-2-iodovinyl phenyl ether **168**. The electron acceptor can only be provided by the addition of phenol to the EBXs since an EDA complex cannot be directly formed from the original substrates, which means that the effect of the two-component ratio of the EDA complex must be taken into account.

**Scheme 61:**
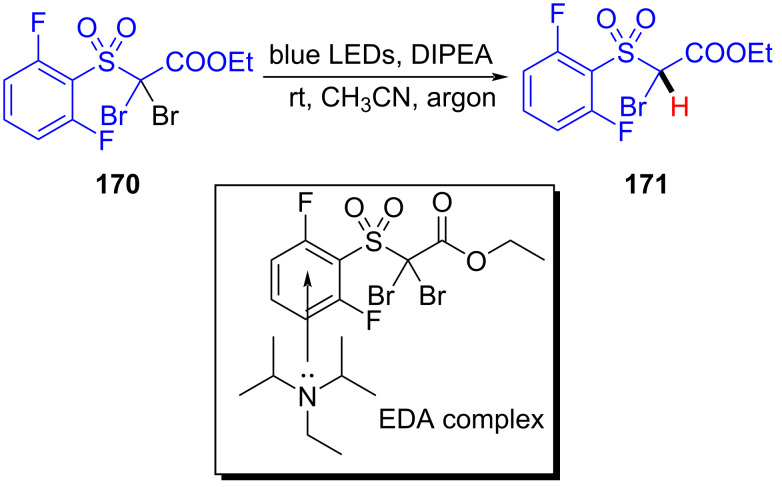
Dehalogenation reaction initiated by an EDA complex.

In 2019, Rathnayake and Weaver, III [37] designed a method of visible-light-promoted EDA-complex-mediated dehalogenation of haloalkanes **170**. The dehalogenation product **171** was afforded based on the presence of the EDA complex formed by DIPEA and haloalkanes **170** under blue-light irradiation (Scheme 61). It was worth mentioning that longer reaction times and increased DIPEA loading were required owing to the inactivity of α-bromoketones, esters, and nonactivated sulfones; however, corresponding products could be given in high yield. When the DIPEA molarity was double that of haloalkanes, the highest yield was given. Since a marked yellow color appeared immediately upon mixing substrates, the existence of an EDA complex could be confirmed by UV–vis spectroscopy.

## Conclusion

In this review, reactions and mechanisms of EDA complexes were discussed from the aspects of cyclization reactions, C–C-, C–S-, C–B-, C–N-, C–P-, C–O-, and C–H bond formations. The absence of transitional-metal catalysts and photosensitizers is the most profound feature of EDA-complex- mediated reactions in most cases. On the other hand, the reaction conditions are mild, and light is utilized as the only external energy source, which is consistent with the theme of green chemistry. However, the comprehension of EDA complexes was established relatively late, mainly owing to the fact that the formation of EDA complexes was regarded as a unique chemical reaction rather than a branch of photochemistry; in addition, for the sake of avoiding BET processes, reactions involving EDA complexes require substrates with corresponding leaving groups, which also significantly limits the development of EDA complexes. In conclusion, although the research on EDA complexes is still in the initial stage, with many challenges to be solved in response, there is no doubt that the future of green chemical synthesis will surely have a very wide prospect for this strategy.
